# Pan-cancer analysis identifies APOC1 as a TAM-derived modulator of adaptive immune resistance and predictor of therapeutic response

**DOI:** 10.1007/s12672-026-05515-x

**Published:** 2026-06-25

**Authors:** Yang Yang, Leilei Fan

**Affiliations:** 1 Department of Thoracic Lymphoma, The Central Hospital of Enshi Tujia and Miao Autonomous Prefecture, Enshi, 445000 Hubei China; 2 Department of Gastrointestinal Surgery, The Central Hospital of Enshi Tujia and Miao Autonomous Prefecture, Enshi, 445000 Hubei China

**Keywords:** *APOC1*, Pan-cancer, Tumor-associated macrophages, Adaptive immune resistance, Genomic instability, Biomarker, Immunotherapy, Pharmacogenomics

## Abstract

**Background:**

Apolipoprotein C1 (*APOC1*) has been implicated in several malignancies, yet its expression patterns, clinical significance, and immunomodulatory roles across cancer types remain poorly characterized.

**Methods:**

We performed a comprehensive multi-omic analysis of *APOC1* across 33 cancer types integrating transcriptomic, proteomic, genomic, epigenomic, and pharmacogenomic data from TCGA, GTEx, CPTAC, and multiple independent external cohorts. Immune infiltration was assessed using seven complementary algorithms. Spatial transcriptomics and single-cell RNA sequencing were employed to determine the cellular source of *APOC1* expression.

**Results:**

*APOC1* upregulation in most cancers was associated with cancer type-specific prognosis. After adjustment for clinical covariates and macrophage infiltration, high APOC1 remained an independent adverse factor in KIRC, LGG, and STAD. *APOC1* expression positively correlated with genomic instability hallmarks, including homologous recombination deficiency and aneuploidy, with these associations largely independent of immune infiltration; in contrast, associations with tumor mutational burden were substantially confounded by macrophage abundance. Immune infiltration analysis revealed a pattern consistent with adaptive immune resistance: *APOC1* correlated positively with immune-activating signatures (STAT1, MHC-II, TCR signaling) and immunosuppressive M2 macrophages and Tregs, yet negatively with anti-tumor effectors (activated NK cells, dendritic cells). Spatial transcriptomics and single-cell RNA sequencing identified tumor-associated macrophages (TAMs) as the primary cellular source of *APOC1*, with transcripts co-localizing with CD68 in tissue sections. *APOC1* expression correlated with multiple immune checkpoint molecules and was elevated in responders to immune checkpoint blockade, consistent with an inflamed yet regulated tumor microenvironment. Pharmacogenomic analyses revealed that APOC1-high tumors display distinct drug response profiles, characterized by resistance to MAPK pathway inhibitors and potential sensitivity to the HDAC inhibitor Entinostat.

**Conclusion:**

This pan-cancer analysis establishes *APOC1* as a context-dependent biomarker and a TAM-derived modulator of adaptive immune resistance, with prognostic and therapeutic implications across malignancies. *APOC1*-expressing TAMs represent a potential target for combination immunotherapy strategies.

**Supplementary Information:**

The online version contains supplementary material available at https://doi.org/10.1007/s12672-026-05515-x.

## Introduction

Cancer remains a leading cause of death worldwide, with projections of over 35 million new cases by 2050 [1, 2], underscoring the need to identify novel therapeutic targets. Among emerging players is apolipoprotein C1 (*APOC1*), a small lipoprotein primarily synthesized in the liver. Traditionally known for regulating lipid metabolism via cholesteryl ester transfer protein inhibition and lipoprotein modulation [3, 4], *APOC1* has recently been implicated in inflammation and immune regulation—processes central to cancer progression [5, 6]. Structurally, its amphipathic helices enable diverse protein-protein interactions that may extend its functions beyond metabolism [7].

Accumulating evidence positions *APOC1* as a putative oncogene in multiple malignancies. Elevated *APOC1* expression promotes proliferation, migration, invasion, and epithelial-mesenchymal transition while suppressing apoptosis, correlating with poor prognosis across cancers including colorectal, breast, glioma, and others [8–11]. Mechanistically, *APOC1* activates MAPK, STAT3, and Wnt/β-catenin pathways and participates in regulatory networks involving lncRNAs and miRNAs [8, 10, 12, 13]. These observations establish *APOC1* as a key player in tumor progression, yet its broader role across cancer types remains incompletely characterized.

The tumor immune microenvironment critically influences cancer outcomes [14]. Recent studies have highlighted *APOC1’s* immunomodulatory functions: in hepatocellular carcinoma, *APOC1* is highly expressed in tumor-associated macrophages (TAMs), and its inhibition promotes M2-to-M1 macrophage polarization via ferroptosis-related mechanisms, enhancing anti-PD1 efficacy [15]. Similarly, in colorectal cancer metastasis, macrophage-derived *APOC1* drives metastasis through the STAT3-CCL2/CCL5 axis [16]. These findings suggest *APOC1’s* pro-tumorigenic effects may be mediated through immune modulation, but a systematic pan-cancer analysis of its immunologic role is lacking.

**Fig. 1:**
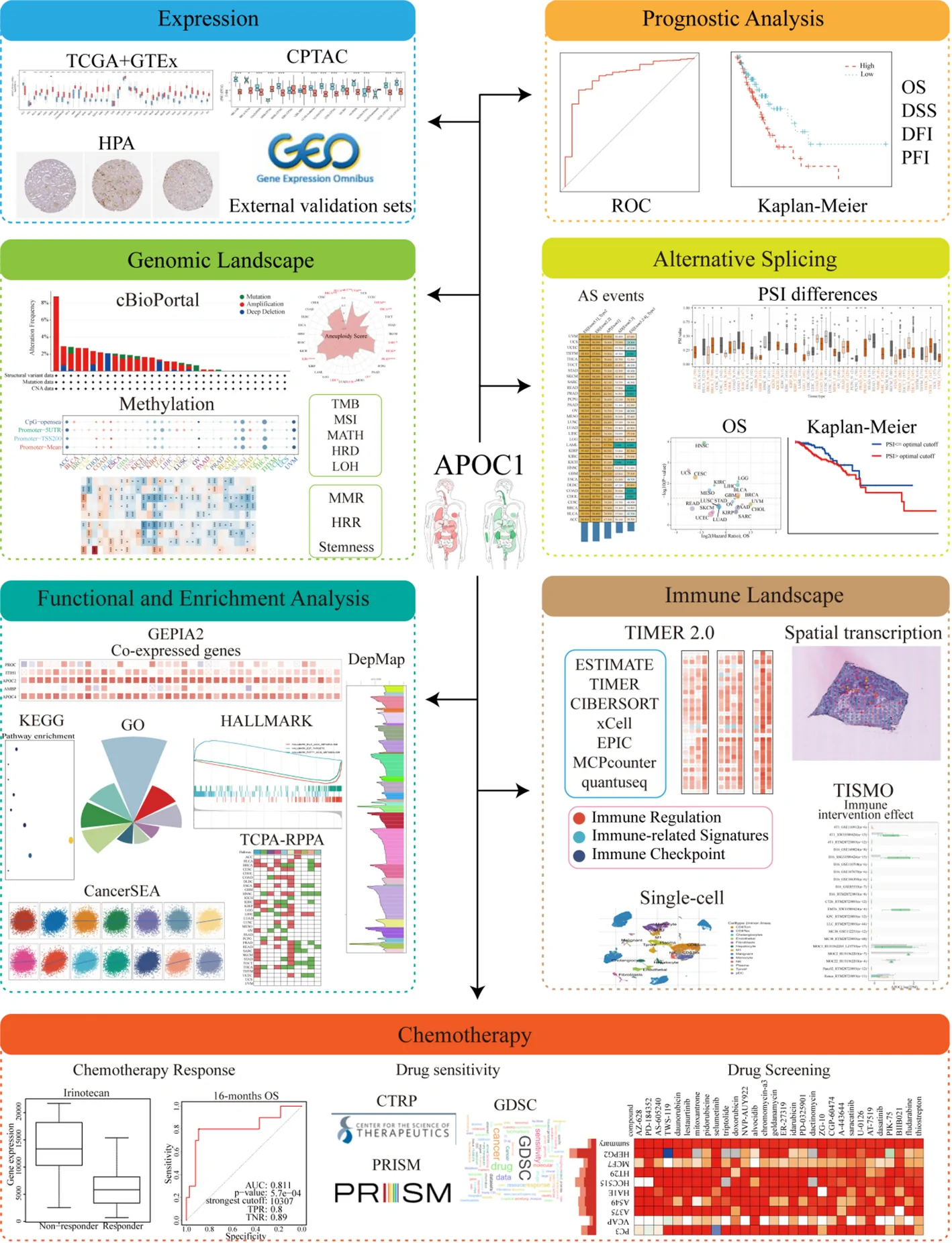
Study flowchart

A recent pan-cancer analysis by Ren et al. characterized *APOC1* expression and its association with M2 macrophage polarization [17]. Although that study provided single-cell evidence for macrophage expression, it lacked spatial transcriptomic validation and did not explore relationships with genomic instability, stemness, or pharmacogenomic vulnerabilities. Notably, all correlation analyses in that work were unadjusted, leaving the possibility that some reported associations were confounded by the very macrophage infiltration that *APOC1* marks. To address these outstanding questions, we performed an integrative multi-omic analysis across 33 cancer types, incorporating spatial transcriptomics, CPTAC proteomics, systematic genomic instability profiling, and pharmacogenomic characterization (Fig. 1).

## Materials and methods

### Data acquisition and preprocessing

Transcriptomic data and matched clinical information for 33 cancer types were obtained from The Cancer Genome Atlas (TCGA) and the Genotype-Tissue Expression (GTEx) project via the UCSC Xena platform (https://xenabrowser.net/). TCGA tumor and GTEx normal samples were integrated and batch-corrected using ComBat. Expression data were log2(TPM + 0.001) transformed. Protein-level expression data were accessed from the Clinical Proteomic Tumor Analysis Consortium (CPTAC) confirmatory datasets via the UALCAN database (http://ualcan.path.uab.edu/). Immunohistochemistry (IHC) images were sourced from the Human Protein Atlas (HPA) database (https://www.proteinatlas.org/). Genomic alteration data, including mutations, copy number variations, and DNA methylation data, were acquired from the cBioPortal for Cancer Genomics (https://www.cbioportal.org/), corresponding to the TCGA PanCancer Atlas studies. For external validation of *APOC1’s* prognostic value, we systematically collected gene expression microarray and RNA-seq datasets with corresponding clinical information from multiple publicly available sources. These included the Gene Expression Omnibus (GEO) database (https://www.ncbi.nlm.nih.gov/geo/), the ArrayExpress database under accession E-MTAB-8588, the Chinese Glioma Genome Atlas (CGGA, http://www.cgga.org.cn/) for CGGA-301 and CGGA-325 cohorts, the International Cancer Genome Consortium (ICGC) data portal for the ICGC-LIRI-JP cohort, and the DKFZ Heidelberg brain tumor cohort (DKFZ2018). A comprehensive list of all external validation cohorts, including accession numbers, sample sizes, cancer types, and platforms, is provided in Table S1.

### Pan-cancer expression, clinicopathological correlation, and prognostic analysis of *APOC1*

To map the pan-cancer expression landscape of *APOC1*, we compared its mRNA levels between tumor and normal tissues, including a paired analysis of TCGA samples with matched adjacent normal tissue. Differential expression at the protein level was validated using CPTAC data, and IHC images from HPA were used to visually assess protein localization and intensity. The association between *APOC1* expression and tumor TNM stage was evaluated using the Kruskal-Wallis test.

Diagnostic utility was assessed by generating receiver operating characteristic (ROC) curves and calculating the area under the curve (AUC) using the R package pROC (version 1.18.0). The correlation between *APOC1* expression and patient survival—including overall survival (OS), disease-specific survival (DSS), disease-free interval (DFI), and progression-free interval (PFI)—was evaluated using univariate Cox proportional hazards regression models. Survival differences between *APOC1* high- and low-expression groups were compared using the log-rank test. The optimal cutoff for each cancer type and endpoint was determined using the maximally selected log-rank statistic. To correct for the multiple testing inherent in data-driven cutoff selection, permutation tests (10,000 iterations) were performed. Multivariate Cox regression was performed for cancer types with significant permutation-adjusted log-rank results and sufficient clinical annotation. Two nested models were constructed: a base model adjusted for age, gender, TNM stage (III/IV vs. I/II), and histological grade (G3/G4 vs. G1/G2); and a full model additionally adjusted for M2 macrophage infiltration (CIBERSORT). The attenuation of the *APOC1* hazard ratio after macrophage adjustment was quantified. The proportional hazards assumption was verified by Schoenfeld residuals (all *P* > 0.05). Cancer types with > 20% missing data for key covariates (TNM stage or histological grade) or with fewer than 50 samples were excluded from multivariate analysis. The prognostic value of *APOC1* was further validated in independent GEO datasets.

###  Genomic alterations of *APOC1* and association with genomic markers

The mutation spectrum and copy number variation (CNV) frequencies of *APOC1* were analyzed using cBioPortal. The impact of *APOC1* CNV on patient prognosis was further assessed using the Copy_Number module of the CIDE database (https://cide.ccr.cancer.gov) with Kaplan-Meier analysis. To investigate the link between *APOC1* and genomic instability, tumor mutational burden (TMB) was calculated for each sample using the R package maftools (version 2.12.0). Precomputed scores for microsatellite instability (MSI), mutant-allele tumor heterogeneity (MATH), homologous recombination deficiency (HRD), loss of heterozygosity (LOH), tumor ploidy, and aneuploidy were obtained from a previously published study [18]. Associations between *APOC1* expression and these genomic features were assessed by Spearman correlation. To distinguish *APOC1*-specific signals from macrophage-driven confounding, partial Spearman correlations controlling for M2 macrophage infiltration (CIBERSORT) were computed; substantial attenuation after adjustment indicated confounding.

### Molecular characterization and regulatory network analysis

To dissect the epigenetic regulation of *APOC1* and its link to genomic stability and stemness, we first downloaded HM450K methylation beta-values for TCGA pan-cancer samples from cBioPortal. For the TSS1500, TSS200, first exon, and 5’UTR regions of the *APOC1* gene, the median beta-value of all CpG sites within each region was calculated as a representative methylation level. Spearman correlation was then used to assess the relationship between regional methylation and *APOC1* expression. The CIDE database’s methylation module was utilized to analyze the correlation between *APOC1* promoter methylation and cytotoxic T lymphocyte infiltration. The correlation between *APOC1* expression and four DNA methyltransferases was also evaluated using Spearman analysis.

To explore the link between *APOC1* and genomic stability maintenance, we analyzed the expression correlation of *APOC1* with a set of DNA mismatch repair (MMR) genes (*MLH1*, *MSH2*, *MSH6*, *PMS2*, *EPCAM*) and a set of homologous recombination repair (HRR)-related genes. Furthermore, we curated 44 epitranscriptomic modifiers associated with m¹A, m⁵C, and m⁶A to investigate their expression correlation with *APOC1*.

Given that tumor stemness is closely linked to therapy resistance, we evaluated the association of *APOC1* with stemness characteristics. Precomputed stemness scores for TCGA pan-cancer samples, including the mRNA expression-based stemness index (RNAss), the epigenetic regulation-based RNA stemness index (EREG.EXPss), and DNA methylation-based indices (DNAss, EREG-METHss, DMPss, ENHss), were obtained from Malta et al. [19]. Spearman correlations assessed relationships between *APOC1* and these stemness scores; partial Spearman correlations controlling for M2 macrophage infiltration distinguished *APOC1*-specific signals from immune infiltration confounds.

### Alternative splicing analysis of *APOC1*

Alternative splicing (AS) events for *APOC1* across all cancer types were obtained from the TCGASpliceSeq database (https://bioinformatics.mdanderson.org/TCGASpliceSeq/). The most frequent AS events were identified using the OncoSplicing platform. Changes in the percent spliced index (PSI) between tumor and normal tissues were compared (|ΔPSI| > 0.1, FDR < 0.05), and the prognostic impact of the PSI value on patient survival was evaluated using Kaplan-Meier analysis.

### Functional enrichment and co-expression network analysis

To explore the biological functions of *APOC1*, we first utilized the Comppi database (https://comppi.biolab.si/) to identify proteins with protein-protein interactions (PPI) with *APOC1*, subsequently filtering for those sharing a common subcellular localization. Concurrently, the GEPIA2 database (http://gepia2.cancer-pku.cn/) was employed to identify the top 100 genes most correlated with *APOC1* expression in a pan-cancer setting. Gene Ontology (GO) and Kyoto Encyclopedia of Genes and Genomes (KEGG) pathway enrichment analyses were performed on this *APOC1*-related gene set using the R package clusterProfiler (version 4.8.0). Furthermore, based on *APOC1* expression levels (high vs. low, defined by tertiles), Gene Set Enrichment Analysis (GSEA) was conducted using the Hallmark and KEGG gene set collections from the MSigDB database.

To decipher APOC1-related functional states, we obtained 14 cancer functional state gene sets from the CancerSEA database. For each sample, a combined z-score was calculated using the z-score algorithm implemented in the GSVA package, reflecting the activity of each functional state. The resulting scores were further standardized using the scale function. Pearson correlation was then computed between APOC1 expression and each standardized functional state score. Reverse-phase protein array (RPPA) data and precomputed activity scores for 10 cancer-related pathways [20] were downloaded from the TCPA database (https://www.tcpaportal.org/). The Wilcoxon rank-sum test was used to compare pathway activity between APOC1 high and low expressors (median split). Genome-wide CRISPR screening dependency scores, corrected by the CERES algorithm, were downloaded from the DepMap portal (https://depmap.org/portal/download/). The *APOC1* CERES score was analyzed for each cell line, with lower scores indicating a stronger inhibitory effect upon *APOC1* knockout.

### Systematic analysis of *APOC1* and the tumor immune microenvironment

To elucidate the immunomodulatory role of *APOC1*, we correlated its expression with 68 immune-related signatures, 60 immune checkpoint molecules, and the abundance of 21 immune cell types. Immune cell infiltration was estimated using a multi-algorithm approach, integrating ESTIMATE, TIMER, CIBERSORT, QUANTISEQ, MCP-counter, xCell, and EPIC. Relationships were assessed by Spearman correlation and visualized with ComplexHeatmap (v2.14.0).

To validate key findings at spatial resolution, we analyzed pan-cancer spatial transcriptomics data from two publicly available resources: SpatialTME (https://www.spatialtme.yelab.site/) [21] for macrophage-enriched region analysis, and SpatialDB (https://www.spatialomics.org/SpatialDB/) [22] for APOC1-CD68 co-localization analysis. Single-cell RNA sequencing data were obtained from the TISCH database to confirm the cellular source of *APOC1*. Detailed sample information for all spatial transcriptomic specimens analyzed is provided in Table S2. Finally, using the TISMO database (http://tismo.cistrome.org/), we compared *APOC1* expression levels in samples before and after cytokine treatment, as well as between responders and non-responders to immune checkpoint blockade (anti-PD-1/anti-CTLA-4).

### Pharmacogenomic analysis of *APOC1* as a potential therapeutic target

To evaluate the predictive value of *APOC1* for treatment response, we utilized the ROC Plotter database (https://www.rocplotter.com/) to compare *APOC1* expression in responders versus non-responders to various chemotherapeutic and targeted therapies. To identify compounds potentially active against *APOC1*-high tumors, we queried the *APOC1*-high gene signature against the Connectivity Map (CMap) database. The primary mechanisms of action (MoA) of the top-scoring compounds were then analyzed. Finally, four pharmacogenomic databases—CTRP, GDSC1, GDSC2, and PRISM—were integrated to systematically analyze the correlation between *APOC1* expression and compound sensitivity.

### Statistical analysis

All statistical analyses were performed using R software (version 4.2.0). Comparisons between two groups were conducted using the Wilcoxon rank-sum test, while comparisons among multiple groups were performed using the Kruskal-Wallis test. Correlation analyses were based on Spearman’s rank correlation coefficient. Survival analyses were conducted using the Kaplan-Meier method with the log-rank test, as well as univariate Cox proportional hazards regression models. Two sources of multiple testing were controlled: (i) cross-cancer comparisons using Benjamini-Hochberg false discovery rate correction; (ii) within-cancer type I error from data-driven optimal cutoffs using 10,000 permutation tests. All tests were two-sided, with adjusted *P* < 0.05 considered significant.

## Results

### *APOC1* is upregulated in pan-cancer and exhibits context-dependent prognostic value

**Fig. 2:**
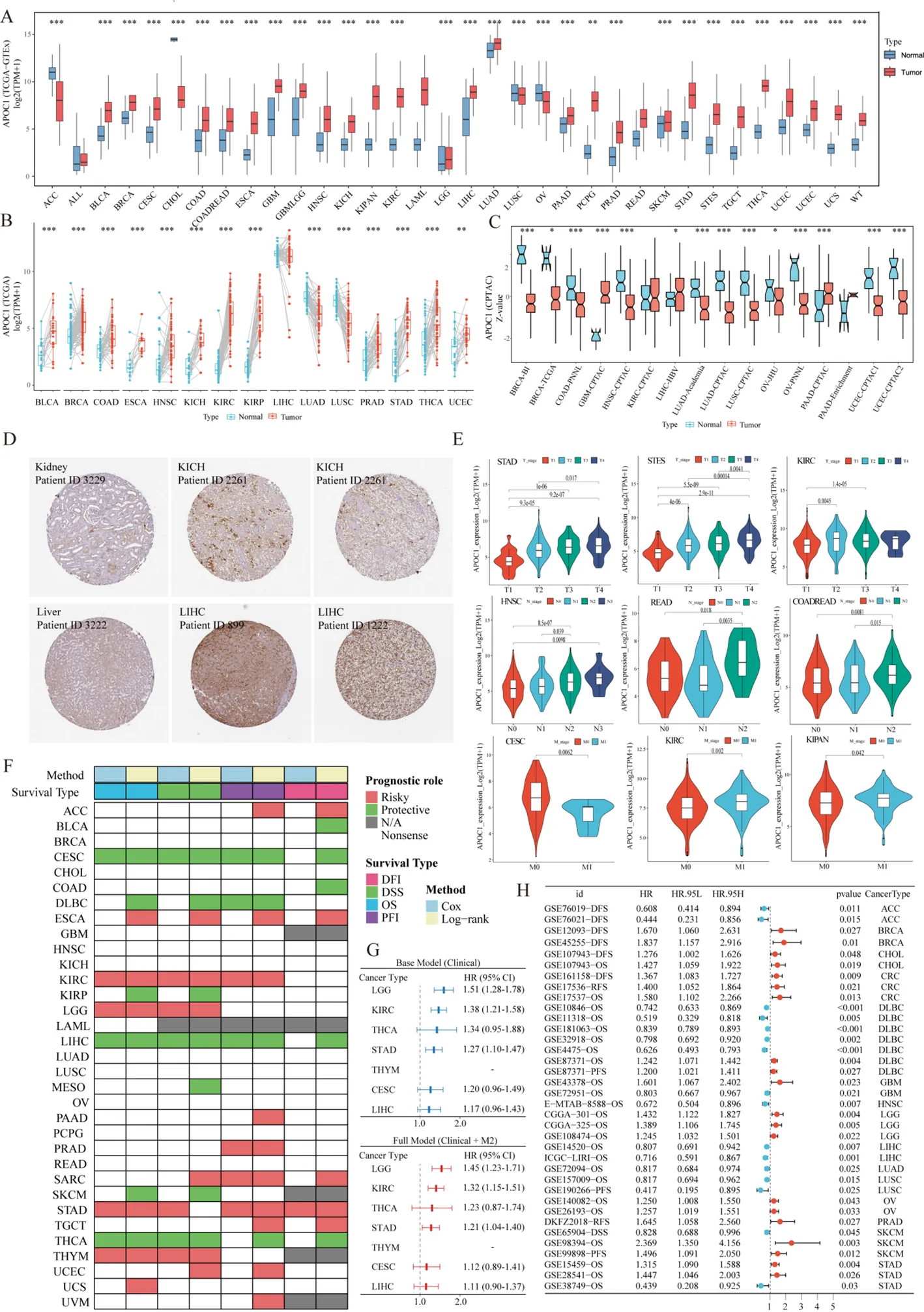
Pan-cancer expression landscape, prognostic value, and multivariate independence of *APOC1*. **A** *APOC1* mRNA expression in tumor versus normal tissues across 33 cancer types (TCGA and GTEx). **B** Paired comparison of *APOC1* mRNA expression in 15 TCGA cancer types with matched adjacent normal samples. **C** APOC1 protein expression in tumor versus normal tissues from CPTAC. **D** Representative immunohistochemistry images from the Human Protein Atlas. **E** Association of *APOC1* mRNA expression with TNM stage. **F** Forest plots of univariate Cox regression for *APOC1* and OS, DSS, DFI, and PFI. **G** Forest plot of multivariate Cox regression comparing the hazard ratio of APOC1 in a base model (clinical covariates) versus a full model (base + M2 macrophage infiltration) for the seven evaluable cancer types. Attenuation of the HR after macrophage adjustment indicates confounding. **H** External validation of APOC1*’*s prognostic value for overall survival across independent cohorts. *P* < 0.05, **P < 0.01, ***P < 0.001

*APOC1* mRNA was significantly upregulated in 28 of 33 cancer types compared to normal tissues, with downregulation confined to ACC and CHOL (Fig. 2A). This upregulation was validated in paired analysis of 15 tumor types with available adjacent normal tissues (Fig. 2B). Protein-level differences were corroborated by CPTAC data: APOC1 protein was markedly elevated in GBM, LIHC, and PAAD, yet reduced in several cancers including BRCA and COAD (Fig. 2C), suggesting post-transcriptional regulation. IHC images from HPA visually confirmed this pattern, showing intense *APOC1* staining in tumor versus normal tissues (Fig. 2D). Furthermore, *APOC1* expression significantly and positively correlated with TNM stage across multiple cancer types (Fig. 2E), implicating its role in tumor progression.

ROC curve analysis indicated robust diagnostic power for several cancers (Fig. S1). APOC1’s prognostic role was cancer type-dependent: high expression indicated poor OS in KIRC, LGG, STAD, and THYM, but favorable OS in CESC, LIHC, and THCA, with similar patterns for DSS, DFI, and PFI (Fig. 2F, S2). Optimal cutoff selection and multiple testing correction. Optimal cutoffs for *APOC1* expression were determined for each cancer type and endpoint using the maximally selected log-rank statistic, and permutation tests (10,000 iterations) were performed to adjust for the multiplicity inherent in this data-driven approach. After permutation adjustment, high *APOC1* expression was significantly associated with overall survival in 11 cancer types: DLBC, KIRC, LGG, LIHC, SKCM, CESC, THCA, ESCA, KIRP, STAD, and THYM (Table S3). Multivariate analysis confirms independent prognostic value of *APOC1*. Of the 11 cancer types with significant permutation-adjusted OS associations, 7 had sufficient clinical annotation for multivariate Cox regression. After adjusting for clinical covariates and M2 macrophage infiltration, high *APOC1* expression remained an independent adverse prognostic factor in KIRC, LGG, and STAD. In CESC, LIHC, and THCA, the univariate associations were attenuated and no longer significant after adjustment (Table S4), indicating confounding by macrophage infiltration. M2 macrophage score itself was independently prognostic in these cancers. Model comparison confirmed that inclusion of M2 infiltration reduced the *APOC1* effect size across all cancer types (Fig. 2G). This prognostic value was validated across independent external cohorts encompassing 15 cancer types (Fig. 2H).

### *APOC1* expression is broadly associated with genomic instability

**Fig. 3:**
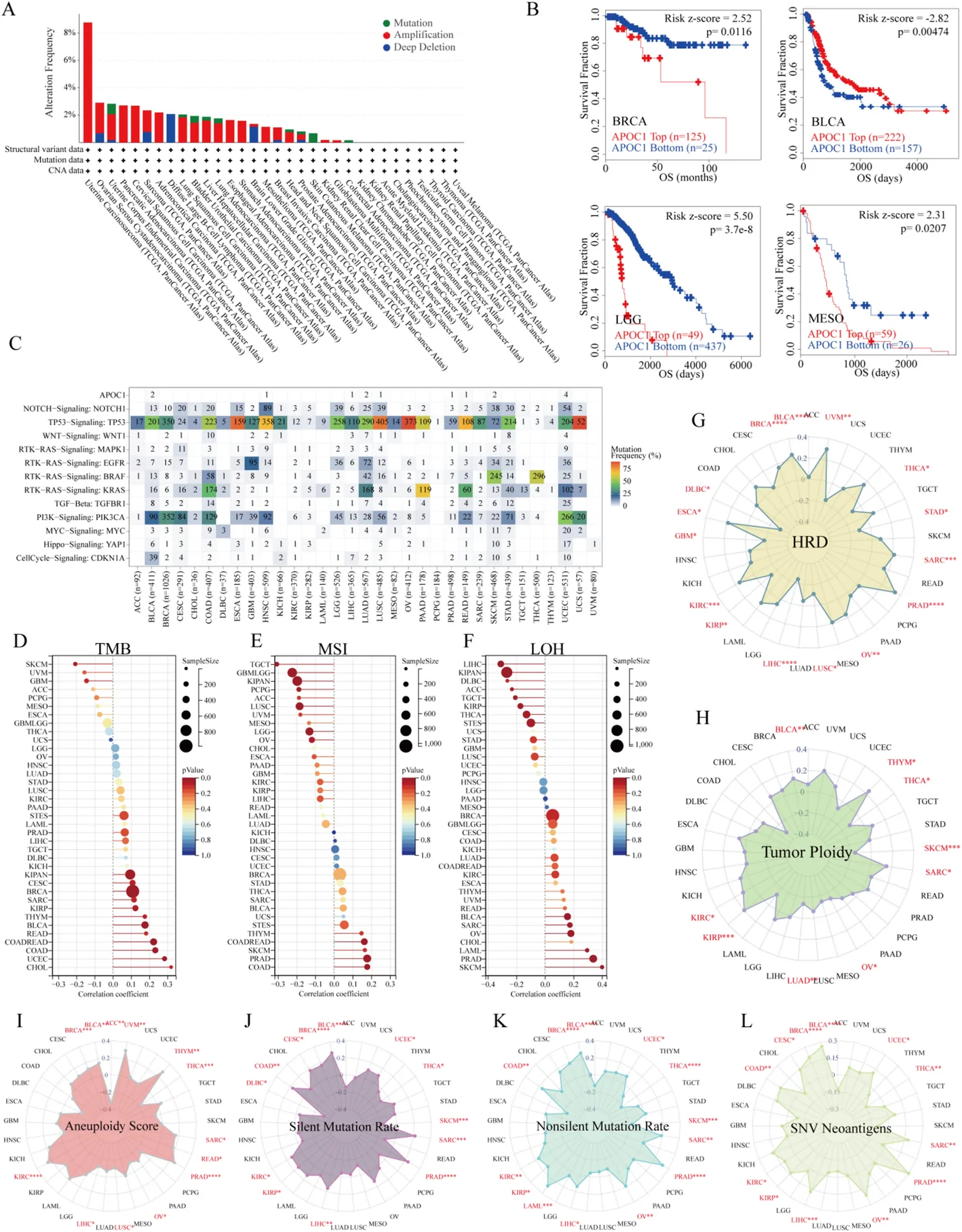
Association of *APOC1* Expression with Genomic Instability Across Cancers. **A** Genetic alteration profile of *APOC1* in TCGA pan-cancer cohorts (cBioPortal). **B** Kaplan**–**Meier curves of overall survival stratified by *APOC1* CNV status in BLCA, BRCA, LGG and MESO. **C** Oncoprint of *APOC1* mutations and co-occurring alterations in key oncogenic pathways. **D–F** Lollipop plots of Spearman correlation between *APOC1* expression and TMB (**D**), MSI (**E**) and LOH (**F**) across cancer types. **G–L** Radar charts of Spearman correlation between *APOC1* expression and HRD (**G**), tumor ploidy (**H**), aneuploidy score (**I**), silent mutation rate (**J**), non-silent mutation rate (**K**), and SNV (**L**). * *p* < 0.05, ** *p* < 0.01, *** *p* < 0.001

cBioPortal data revealed widespread *APOC1* copy number variations across cancers, including high-frequency amplification in UCS and OV, and deep deletion in DLBC and LGG (Fig. 3A). Stratified analysis based on CNV status demonstrated that high CNV was associated with better survival in BLCA but worse survival in BRCA, LGG and MESO subtypes (Fig. 3B). The pan-cancer mutational landscape of *APOC1* in the context of classic oncogenic signaling pathways is illustrated in Fig. 3C.

*APOC1* expression correlated with multiple genomic instability markers in a cancer-specific manner. It correlated positively with TMB in CHOL and UCEC, and with MSI in COAD and PRAD (Fig. 3D**–**E). Notably, *APOC1* showed strong and widespread positive correlations with HRD and LOH scores in most cancer types (Fig. 3F**–**G), suggesting a link to dysregulated DNA damage repair pathways. MATH-based clonal heterogeneity correlated positively with APOC1 in LAML, CESC, SARC, and BLCA, but negatively in CHOL, PAAD, KIPAN, and LIHC (Fig. S3A).

*APOC1* demonstrated highly consistent positive correlations with tumor ploidy and aneuploidy across 33 cancer types (Fig. 3H**–**I). Notably, *APOC1* also exhibited positive correlations with tumor purity in several cancer types (Fig. S3B), an observation that appears paradoxical given its predominant expression in TAMs; partial correlation analysis controlling for M2 macrophage infiltration attenuated or reversed this association in multiple cancer types (Table S5), indicating confounding by the complex relationship between immune infiltration and purity estimation. Neoantigen load associations were tissue-specific, positive in DLBC, CHOL, and COAD, but negative in TGCT and UVM (Fig. S3C). APOC1 expression also correlated strongly with both silent and non-silent mutation rates (Fig. 3J**–**L). A heatmap summarizing APOC1’s extensive associations with immune response features and genomic instability phenotypes is shown in Fig. S3D. To systematically distinguish *APOC1*-specific associations from those driven by macrophage abundance, we performed partial correlation analyses across all genomic instability markers. After adjusting for M2 macrophage infiltration, correlations with HRD, aneuploidy, and LOH remained significant in most cancer types, whereas associations with TMB and MSI were substantially attenuated (Table S5), indicating that *APOC1* is independently linked to structural genomic instability but not to mutational load per se. Collectively, these findings suggest that high *APOC1* expression is closely linked to enhanced genomic instability in tumors.

### *APOC1* is associated with epigenetic regulation, dna repair pathways, and tumor stemness features

**Fig. 4:**
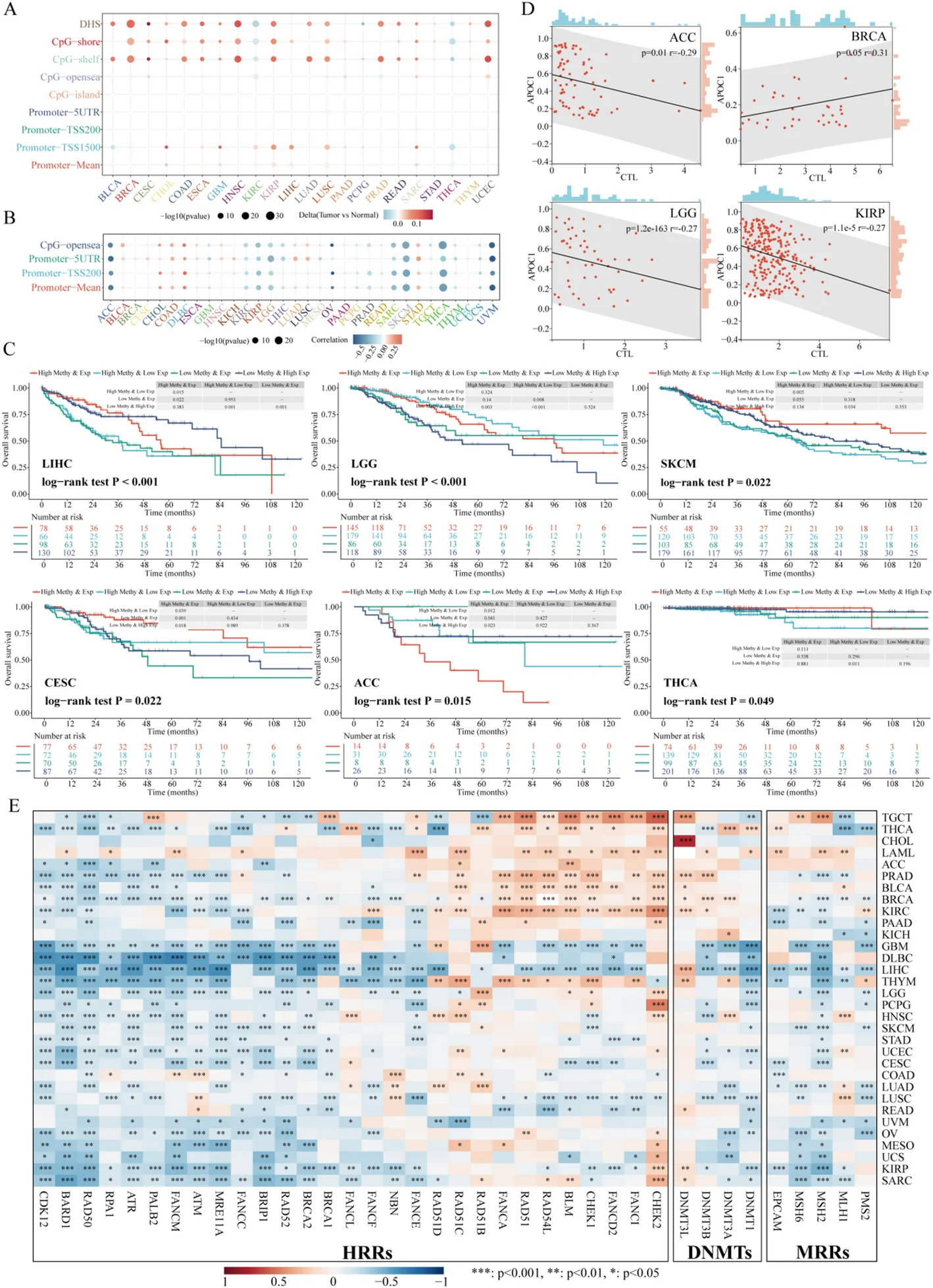
*APOC1* Associations with Epigenetic Regulation, DNA Repair, and Stemness. **A** Bubble plot of *APOC1* methylation differences at genetic loci across cancers. **B** Bubble plot of correlations between *APOC1* expression and methylation at indicated loci. **C** Kaplan-Meier curves for overall survival stratified by *APOC1* promoter methylation status in the indicated cancer types. **D** Scatter plots of *APOC1* promoter methylation versus CTL marker expression. **E** Heatmap of Spearman correlations between *APOC1* and DNMTs, MMR genes, and HRR genes across cancers. *p < 0.05, **p < 0.01, ***p < 0.001

We next investigated *APOC1’s* potential role in epigenetic and DNA repair machinery. APOC1 expression negatively correlated with its promoter methylation across most cancers, with regional specificity (Fig. 4A**–**B, S4A). The most pronounced negative correlations were at TSS1500 and TSS200 regions. High *APOC1* promoter methylation predicted longer survival in LIHC, LGG, SKCM, and CESC (Fig. 4C), suggesting a protective role for methylation-mediated silencing. *APOC1* promoter methylation showed heterogeneous correlations with cytotoxic T lymphocyte infiltration across cancer types (Fig. 4D).

APOC1-DNMT correlations were cancer type-specific, positive in CHOL, PRAD, BRCA, and TGCT, but negative in GBM, DLBC, UCEC, CESC, and LUSC (Fig. 4E). Regarding DNA repair pathways, *APOC1* expression positively correlated with MMR gene expression (MLH1, MSH2, MSH6, PMS2) in MSI-high cancers (COAD, READ, UCEC), but this association was substantially attenuated after adjusting for M2 macrophage infiltration (Table S5), indicating that it largely reflects the elevated immune infiltration characteristic of MSI-high tumors. For HRR genes, *APOC1* correlated positively with BRCA1, BRCA2, and RAD51 expression in HRD-high cancers such as BRCA and OV (Fig. 4E). Although HRD denotes functional repair deficiency, the positive correlation with HRR gene expression at the transcriptional level is not contradictory. Additionally, APOC1 correlated with key RNA methylases (*METTL3*, *FTO*, *ALKBH5*) in certain cancer types (Fig. S4B), suggesting potential involvement in RNA-level epigenetic regulation.

Tumor stemness analysis revealed that *APOC1* strongly and consistently correlated with DNA methylation-based stemness indices (DNAss/EREG-METHss/DMPss/ENHss): negatively in DLBC, KIPAN, LUSC, and KIRC, but positively in OV, TGCT, UVM, and LIHC (Fig. S5A-D). Strikingly, *APOC1* was positively correlated with mRNA-based stemness indices (RNAss and EREG.EXPss) in most cancers (Fig. S5E**–**F). After adjusting for M2 macrophage abundance, the positive correlations with mRNA-based stemness indices persisted in most cancers, whereas DNA methylation-based stemness associations were partially attenuated (Table S6), indicating that the mRNA stemness signal is independent of immune infiltration. Collectively, these findings implicate *APOC1* in epigenetic and transcriptomic regulation, linking it to DNA methylation, RNA modification, DNA repair, and stemness phenotypes, while highlighting that certain stemness associations may reflect the pro-stemness microenvironment shaped by *APOC1*-expressing TAMs.

### Clinically relevant alternative splicing events of *APOC1*

**Fig. 5:**
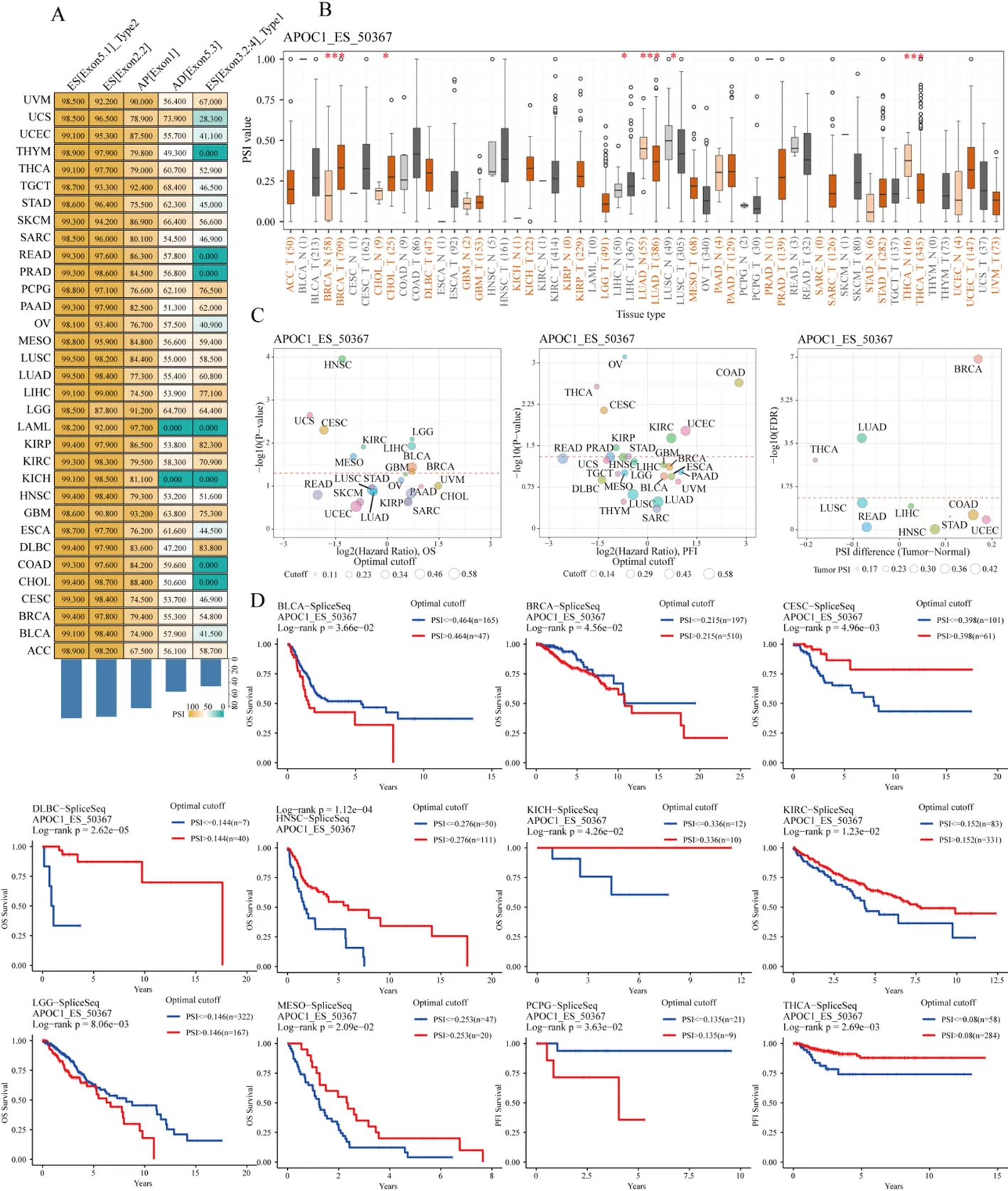
Clinically Relevant Alternative Splicing Events of *APOC1*. **A** Top five *APOC1* alternative splicing events by frequency across TCGA cancers. ES, exon skip. **B** PSI levels of *APOC1*_ES_50367 in pan-cancer, adjacent, and normal tissues. **C** Tumor-adjacent PSI differences and their associations with OS and PFI. Red dashed line, FDR = 0.05; point size, tumor PSI value. **D** Kaplan-Meier curves for *APOC1*_ES_50367 PSI and patient survival. *p < 0.05, **p < 0.01, ***p < 0.001

We analyzed the alternative splicing (AS) landscape of *APOC1* and identified 101 clinically significant AS events (Table S7), with exon skip representing the most frequent event type (Fig. 5A). Taking the two most frequent events (ES_50367, ES_308128) as examples, percent spliced in (PSI) levels differed significantly between tumor and normal tissues. *APOC1*_ES_50367 PSI was significantly higher in BRCA, CHOL, and LIHC tumors, but lower in LUAD, LUSC, and THCA compared to normal tissues (Fig. 5B). Notably, high PSI levels were significantly associated with poor prognosis in BLCA, BRCA, LGG, and PCPG (Fig. 5C**–**D). Results for *APOC1*_ES_308128 are shown in Fig. S6A-C. These findings suggest that aberrant *APOC1* splicing may contribute to malignant progression, with splice variants potentially possessing functions distinct from the full-length transcript.

### Functional enrichment links *APOC1* to immune-inflammatory response and metabolic remodeling

**Fig. 6:**
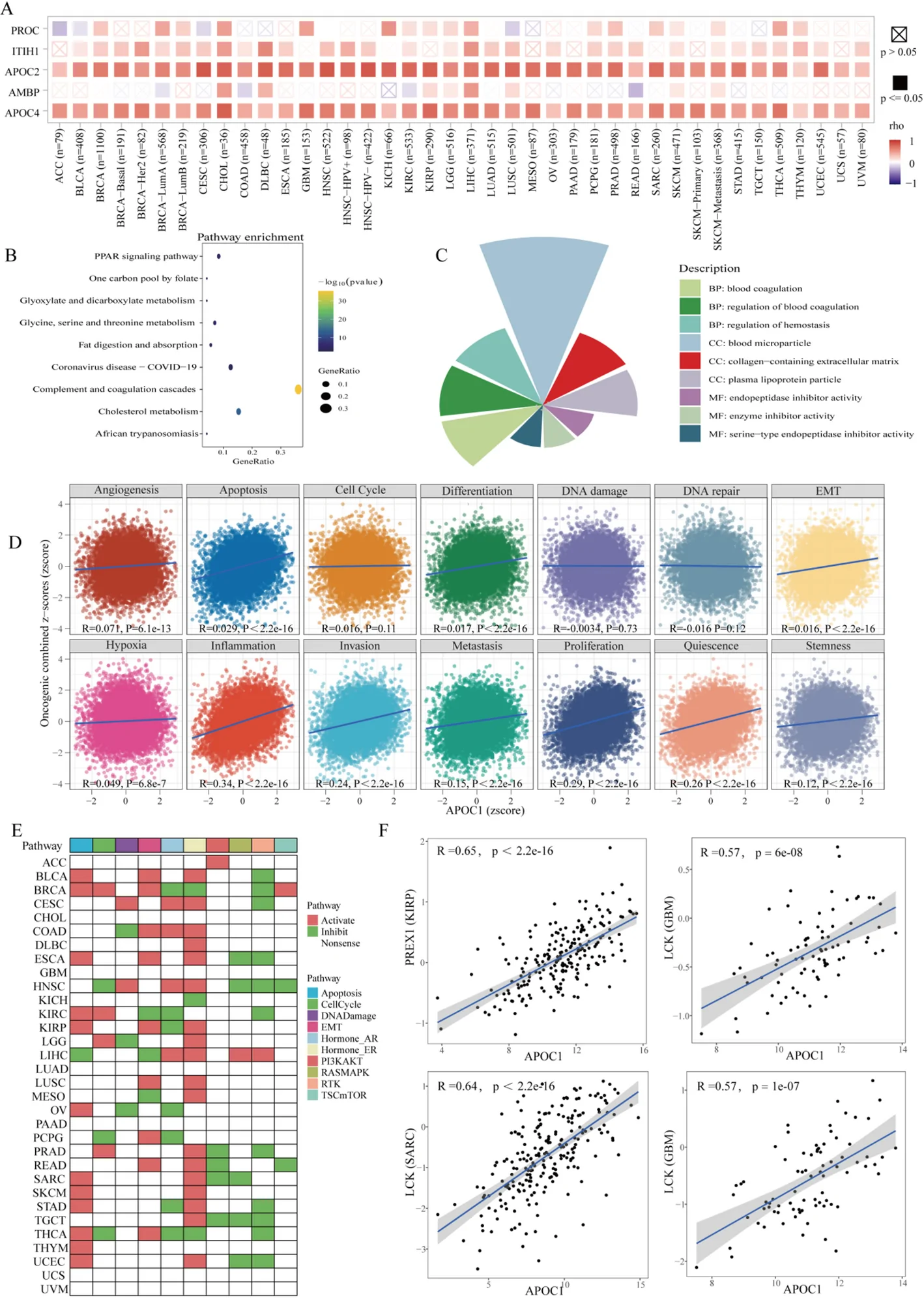
Functional Enrichment and Co-Expression Analysis of *APOC1*. **A** Top five genes co-expressed with *APOC1* (GEPIA2). Heatmap, Spearman correlation coefficients across cancer types. **B–C** KEGG (**B**) and GO (**C**) enrichment analyses of *APOC1*-co-expressed genes. **D** Pearson correlations between *APOC1* expression and cancer functional state scores (CancerSEA). **E** Cancer pathway activity scores (RPPA) in *APOC1*-high versus -low groups. **F** Scatter plots of APOC1 expression versus PREX1 and LCK protein levels (RPPA). *p < 0.05, **p < 0.01, ***p < 0.001

To explore the biological functions of *APOC1*, we constructed a co-expression network and performed functional enrichment analysis. From the Comppi database, 24 interacting proteins sharing common subcellular localization with APOC1 were identified (Fig. S7A). Using GEPIA2, we identified the top 100 genes co-expressed with *APOC1*, with APOC4, AMBP, APOC2, ITIH1, and PROC showing the highest correlation across most cancer types (Fig. 6A). KEGG and GO enrichment analyses of *APOC1*-correlated genes consistently pointed to roles in complement and coagulation cascades, lipoprotein metabolism, and extracellular matrix organization (Fig. 6B–C, Table S8). GSEA demonstrated that samples with high *APOC1* expression were significantly enriched for immune-related Hallmark pathways, including inflammatory response, EMT, interferon-gamma response, and complement (Fig. S7B).

APOC1 correlated positively with EMT, angiogenesis, stemness, and invasion scores, and negatively with apoptosis and differentiation (Fig. 6D), linking APOC1 to an aggressive phenotype. RPPA-based pathway activity analysis showed significantly enhanced activity of apoptosis, EMT, and hormone-related pathways in the *APOC1*-high group (Fig. 6E). Correspondingly, *APOC1* expression positively correlated with protein levels of PREX1 (EMT-related) and LCK (T-cell signaling) (Fig. 6F, Fig. S8A). Finally, DepMap data indicated that *APOC1* knockout exhibited proliferative inhibitory effects (CERES score < 0) in a subset of cancer cell lines, suggesting it may be a dependency gene in specific tumors (Fig. S8B). Collectively, these results link *APOC1* to remodeling of the tumor immune-inflammatory microenvironment, EMT activation, and metabolic reprogramming.

### High *APOC1* expression is associated with an immunosuppressive tumor microenvironment

To elucidate the immunomodulatory role of *APOC1*, we systematically analyzed its relationship with the tumor immune microenvironment. *APOC1* expression displayed a paradoxical immune correlation profile: it was positively associated with markers of active anti-tumor immunity, including STAT1, MHC-II molecules, and T-cell receptor signaling, yet simultaneously exhibited positive correlations with multiple immune checkpoint molecules (PD-L1, CTLA-4) and negative correlations with certain immunosuppressive signatures (TGF-β, IL-8) (Fig. S9A–B). This pattern is characteristic of an adaptive immune resistance state, in which an initial anti-tumor immune response is counterbalanced by compensatory inhibitory mechanisms.

**Fig. 7:**
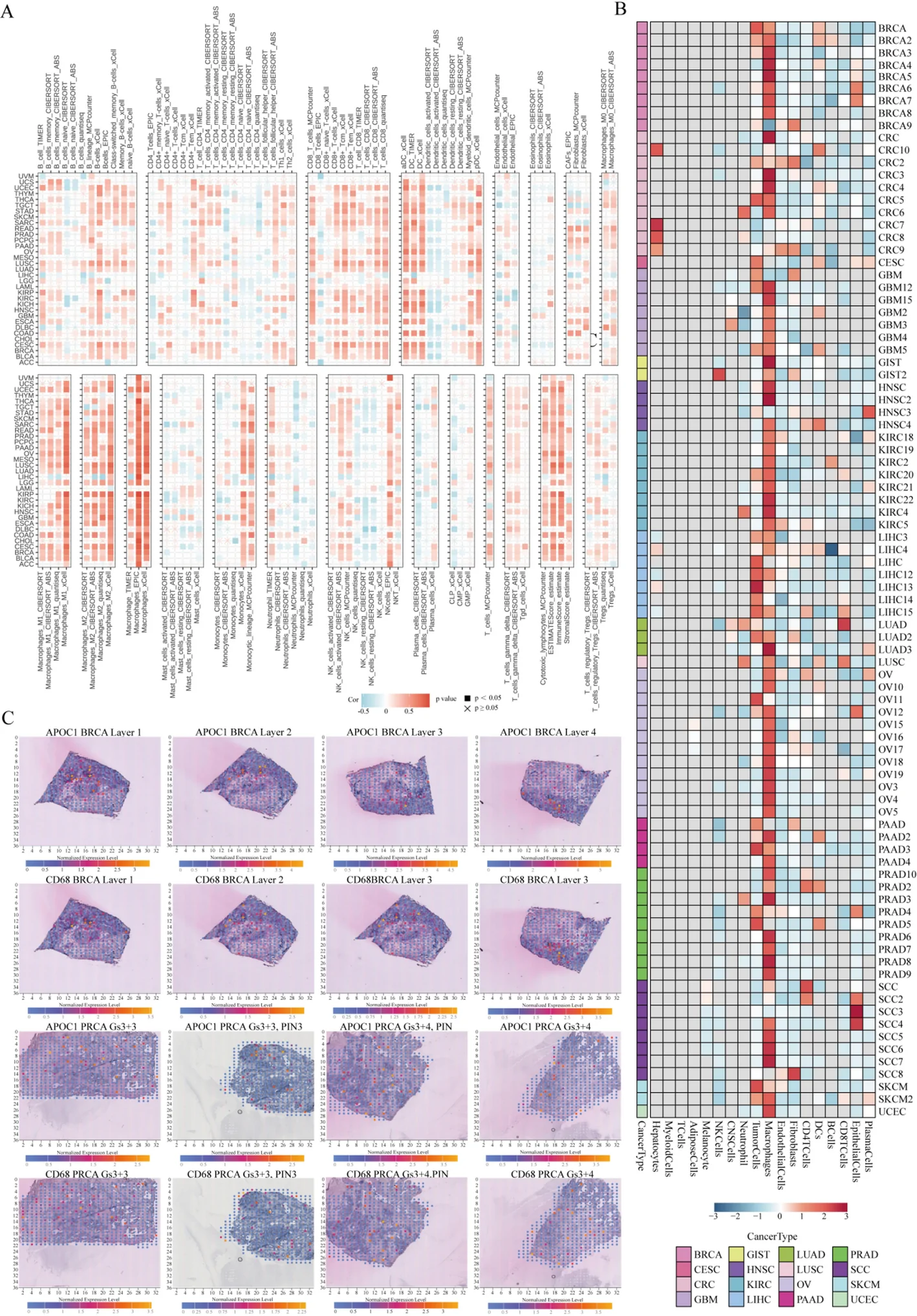
*APOC1* and the immunosuppressive tumor microenvironment. **A** Spearman correlations of *APOC1* expression with TME scores (Stromal, Immune, ESTIMATE) and immune cell abundance estimated by seven deconvolution algorithms. **B** Heatmap of *APOC1* expression across cell-type microdomains in spatial transcriptomics sections (SpatialTME). **C** Spatial co-localization of *APOC1* and CD68 in representative BRCA and PRAD tissue sections (SpatialDB)

This revealed broad positive correlations with M2 macrophages, Tregs, and follicular helper T cells, contrasting with negative correlations with anti-tumor effectors (plasma cells, activated NK cells, dendritic cells) (Fig. 7A). ESTIMATE analysis confirmed that *APOC1*-high tumors exhibited higher immune and stromal scores (Fig. 7A), indicating microenvironment remodeling.

**Fig. 8:**
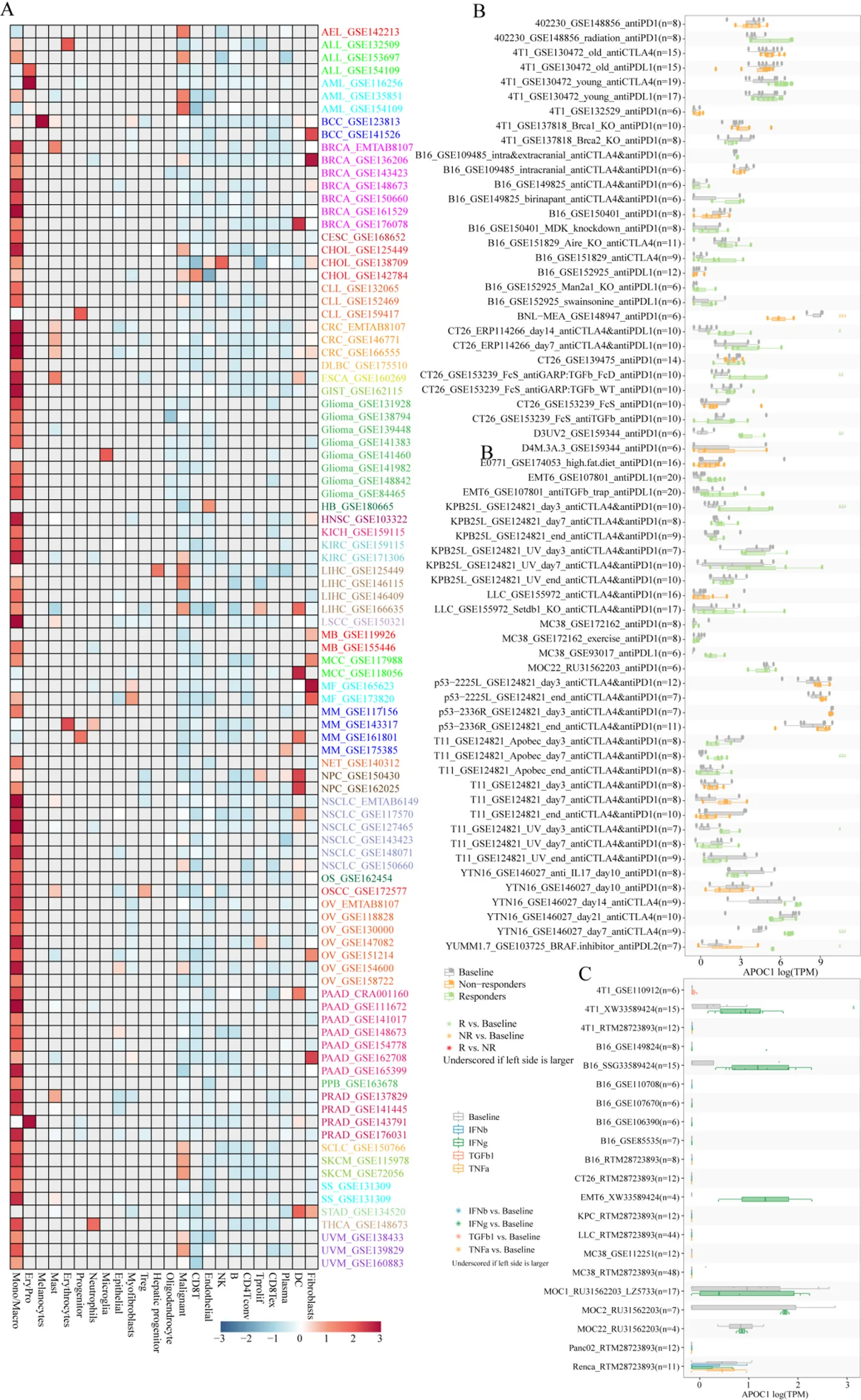
Single-Cell and Immunotherapy Response Analysis of *APOC1*. **A** scRNA-seq analysis of *APOC1* expression across cell types in the TME (TISCH). **B** *APOC1* expression in responders versus non-responders to anti-PD-1/anti-CTLA-4 (TISMO). **C** *APOC1* expression before and after cytokine stimulation (IFN-γ, TNF-α) in vitro. *p < 0.05, **p < 0.01, ***p < 0.001

Analysis of spatial transcriptomics data from the SpatialTME database confirmed that *APOC1* expression was significantly elevated in macrophage-enriched regions across multiple cancer types (Fig. 7B). Furthermore, using the SpatialDB platform, we observed that *APOC1* transcripts co-localized with the macrophage marker CD68 in BRCA and PRAD tissue sections (Fig. 7C). Single-cell RNA sequencing confirmed predominant *APOC1* expression in monocyte/macrophage populations rather than malignant epithelial cells (Fig. 8A). Finally, *APOC1* expression was significantly elevated in responders to immune checkpoint blockade and following cytokine stimulation (Fig. 8B–C), suggesting it may serve as a marker of the underlying inflammatory response or represent a component of a therapy-induced negative feedback loop. Furthermore, *APOC1* expression displayed a tumor-dependent association pattern with immune-related genes (Fig. S10). Thus, APOC1⁺ TAMs mark a state of adaptive immune resistance characterized by concurrent immune activation (STAT1, MHC-II) and compensatory immunosuppression (M2 infiltration, Tregs, checkpoint expression). This dual profile may explain why APOC1-high tumors, despite harboring compensatory immunosuppressive features, can remain responsive to immune checkpoint blockade.

### Pharmacogenomic characterization of *APOC1* as a potential therapeutic target

**Fig. 9:**
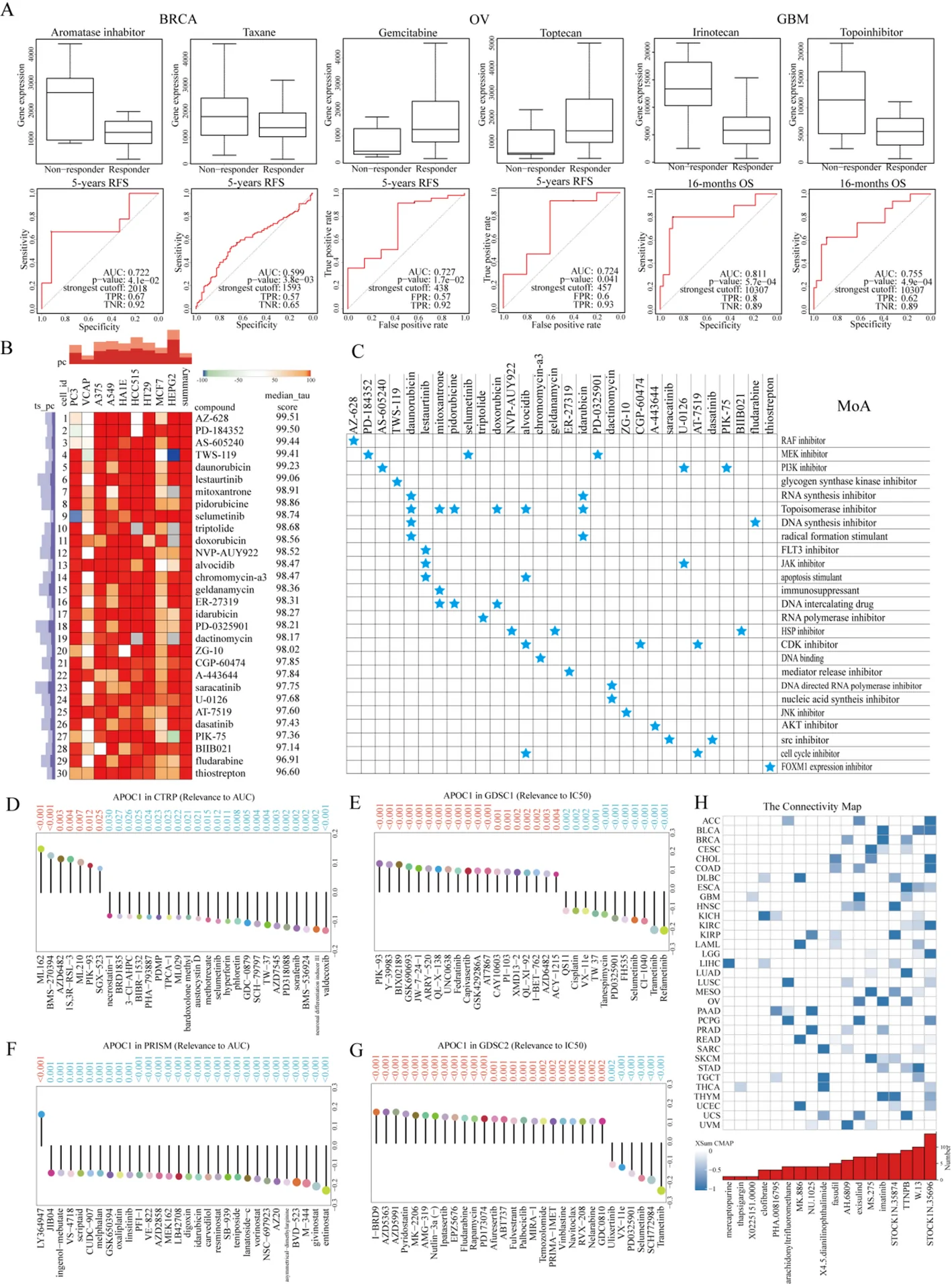
Pharmacogenomic Analysis of *APOC1*. **A** *APOC1* expression in responders versus non-responders to chemotherapy (ROC Plotter). **B** Heatmap of the top 30 candidate compounds (CMap) connected to the *APOC1-high* signature. **C** MoA enrichment analysis of the top 30 compounds. **D–G** Spearman correlations between *APOC1* expression and drug sensitivity in the CTRP (**D**), GDSC1 (**E**), GDSC2 (**F**), and PRISM (**G**) databases. **H** Entinostat (MS-275) as a top compound reversing the *APOC1*-high transcriptomic signature

Based on the potential role of *APOC1* in tumor progression and immune evasion, we evaluated its potential as a therapeutic target and a predictive biomarker. ROC Plotter analysis revealed that *APOC1* expression levels were associated with chemotherapy response in a treatment- and cancer-specific manner. In OV, responders to gemcitabine and topotecan exhibited higher *APOC1* expression. In contrast, in BRCA and GBM, non-responders to multiple regimens showed higher *APOC1* expression (Fig. 9A), suggesting that high *APOC1* expression might be linked to chemoresistance in certain cancers.

Given that patients with *APOC1*-high tumors responded poorly to conventional chemotherapy, we sought to identify compounds with potential activity against these tumors. CMap analysis identified 30 candidate compounds whose signatures were most significantly connected to the high *APOC1* expression signature (Fig. 9B). Enrichment analysis revealed that MAPK signaling pathway inhibitors, particularly MEK inhibitors, and topoisomerase inhibitors were the most prominent classes among these (Fig. 9C). Drug sensitivity analysis across four independent pharmacogenomic databases (CTRP, GDSC1, GDSC2, and PRISM) revealed that *APOC1* expression was significantly negatively correlated with sensitivity to MAPK pathway inhibitors (e.g., trametinib, selumetinib), indicating resistance to these agents in *APOC1*-high tumors. Similarly, negative correlations were observed for topoisomerase II inhibitors. Conversely, *APOC1* expression was positively correlated with sensitivity to PI3K/AKT pathway inhibitors across multiple datasets (Fig. 9D–G). Of particular note, the HDAC inhibitor Entinostat (MS-275) was identified as one of the top compounds capable of reversing the transcriptomic signature driven by high *APOC1* expression (Fig. 9H). Coupled with the previously observed correlation between *APOC1* and epigenetic modifiers, this provides a theoretical rationale for exploring combination therapeutic strategies targeting *APOC1*-high tumors.

## Discussion

The present study provides a comprehensive pan-cancer characterization of *APOC1*, revealing multifaceted roles for this apolipoprotein that extend far beyond its classical function in lipid metabolism. Through integrative analysis of transcriptomic, proteomic, genomic, and pharmacogenomic data across 33 cancer types, we demonstrate that *APOC1* is broadly upregulated in malignancies and exhibits cancer type-specific associations with patient prognosis, genomic instability, epigenetic regulation, and immune modulation. These findings position *APOC1* as a context-dependent biomarker and highlight the complexity of its TME biology.

*APOC1* mRNA is significantly upregulated in most cancers, suggesting a broad role in oncogenesis consistent with reports in colorectal [9], breast [23], and diffuse large B-cell lymphoma [24]. Downregulation confined to ACC and CHOL, coupled with mRNA-protein discordance in BRCA and COAD, implicates post-transcriptional regulation. Such mechanisms may include miRNA-mediated repression or altered protein stability, consistent with evidence that *APOC1* is modulated by epigenetic modifiers and non-coding RNAs, including pseudogene *APOC1P*-derived transcripts [25–27]. *APOC1* exhibited striking cancer type-specific prognostic value: high expression predicted poor overall survival in KIRC, LGG, STAD, and THYM, but favorable outcomes in CESC, LIHC, and THCA. This context-dependent role was validated across independent cohorts, confirming its clinical significance is linked to specific tumor biology. Such heterogeneity may reflect *APOC1’s* predominant expression in tumor-associated macrophages rather than malignant cells, as shown by our single-cell data. Similar observations in colorectal cancer [16] and glioma [10] support this interpretation.

A key finding is the widespread association between *APOC1* and genomic instability hallmarks. Positive correlations with HRD, aneuploidy, and HRR genes (*BRCA1*,* BRCA2*,* RAD51*) suggest *APOC1* reflects impaired DNA repair. This aligns with evidence linking lipid metabolism to genomic stability via redox homeostasis [28] and lipid droplet signaling [29]. More plausibly, given *APOC1’s* predominant expression in macrophages, high levels may signify a macrophage-driven inflammatory microenvironment inducing replicative stress and secondary genomic instability in adjacent malignant cells, consistent with literature documenting macrophage-mediated DNA damage through reactive oxygen species [30–33]. Positive correlations with TMB and MSI in selected cancers further link *APOC1* to mutagenic processes relevant to therapy. Given that high TMB/MSI predict response to immune checkpoint blockade [34–36], *APOC1*-expressing tumors—despite their immunosuppressive microenvironment—may respond to checkpoint inhibition. This aligns with elevated *APOC1* in anti-PD-1 responders, and future studies should explore *APOC1* as a dual biomarker for genomically unstable and immunoresponsive tumors.

How might a macrophage-derived factor influence tumor cell stemness? *APOC1* expression negatively correlates with promoter methylation (TSS1500/TSS200), consistent with canonical transcriptional silencing in cancer [17, 37]. High promoter methylation predicts longer survival in LIHC, LGG, SKCM, and CESC, suggesting epigenetic silencing may confer survival advantage by attenuating protumorigenic functions of *APOC1*-expressing macrophages. Heterogeneous *APOC1* methylation–CTL correlations suggest a role for epigenetic regulation in shaping immune recruitment [38]. Methylation-dependent silencing of immune genes can create immune-excluded phenotypes [39], and *APOC1* methylation may similarly contribute to tumor immune programming. The positive correlation with mRNA-based stemness indices, contrasting with negative associations in DNA methylation-based indices in specific tumors, implicates APOC1 in stem-like phenotypes—a key driver of therapy resistance [40]. Given *APOC1’s* predominant expression in macrophages, TAMs secrete factors that induce cancer stem cell phenotypes via paracrine signaling [41, 42]; TAM-derived IL-6 and TGF-β activate stemness programs in adjacent cancer cells [43, 44]. *APOC1* may directly participate or serve as a surrogate marker of a pro-stemness TAM state. Together with its correlation with EMT and invasion scores, these findings suggest that elevated *APOC1* expression is associated with aggressive tumor phenotypes, potentially reflecting the interplay between TAM-derived factors and tumor cell behavior at the tumor-microenvironment interface.

After multivariate adjustment, *APOC1* remained an independent prognostic factor in KIRC, LGG, and STAD, indicating that its prognostic value in these cancers extends beyond reflecting macrophage abundance. In CESC, LIHC, and THCA, the associations were confounded by M2 macrophage infiltration. This context-dependent independence has translational implications: APOC1 may represent a therapeutic target where it retains independent prognostic significance, and a TAM surrogate marker elsewhere.

A seemingly paradoxical finding was the positive correlation between *APOC1*, a TAM-derived molecule, and tumor purity. We propose three non-mutually exclusive mechanisms for this observation. First, the ESTIMATE algorithm derives purity as a complement of immune and stromal scores; positive correlations between immune markers and inferred purity are a documented computational phenomenon [45], and our partial correlation analyses confirmed that controlling for M2 abundance attenuated the *APOC1*-purity association. Second, highly aneuploid tumors harbor M2-polarized macrophages alongside expanding malignant clones [46]; the persistent *APOC1*-aneuploidy correlation after macrophage adjustment supports a model of aneuploidy-driven co-expansion of malignant cells and *APOC1* + TAMs. Third, distinct TAM subsets occupy segregated niches within the tumor microenvironment, with certain subpopulations enriched in tumor cell-dense regions rather than stromal compartments; our spatial co-localization of *APOC1* with CD68 in tissue sections is consistent with this spatial organization. Together, these mechanisms provide a coherent resolution of the purity paradox, though direct spatial quantification of *APOC1* + TAM density relative to local tumor cell abundance is warranted to determine the relative contribution of each factor.

HRR gene expression in HRD tumors: resolving an apparent paradox. A counterintuitive finding was the positive correlation between *APOC1* and HRR gene expression (*BRCA1*,* BRCA2*,* RAD51*) in HRD-high tumors, given that HRD is defined by functional deficiency in homologous recombination repair. This is resolved by recognizing that genomic HRD status and transcriptional HRR gene expression are distinct biological dimensions: compensatory upregulation of HRR genes in HRD tumors is well-documented. *RAD51* overexpression has been shown to bypass *BRCA1* deficiency in breast cancer by restoring *RAD51* subnuclear assembly independently of *BRCA1* function [47], and HRD-high tumors in TCGA consistently exhibit elevated mRNA levels of multiple HRR pathway members [48]. Mechanistically, accumulated DNA damage in HRD tumors activates the cGAS-STING innate immune pathway, triggering interferon signaling that transcriptionally upregulates DNA repair genes. This provides a potential link to *APOC1*: STING activation in TAMs by tumor-derived cytosolic DNA may simultaneously drive *APOC1* expression and contribute to the inflammatory milieu that upregulates HRR transcription in neighboring tumor cells. In contrast, the positive correlation between *APOC1* and MMR gene expression in MSI-high cancers was substantially attenuated after macrophage adjustment, confirming that this association is driven by the elevated immune infiltration of hypermutated tumors.

Arguably the most compelling findings relate to *APOC1*’s immunomodulatory functions. Integrating seven deconvolution algorithms, we identified a profile most consistent with adaptive immune resistance: APOC1 correlated positively with both immune-activating signals (STAT1, MHC-II, TCR signaling) and immunosuppressive cell types (M2 macrophages, Tregs), yet negatively with anti-tumor effectors (activated NK cells, dendritic cells). This dual signature—concurrent immune activation and compensatory inhibition—aligns with the adaptive immune resistance paradigm, wherein an active anti-tumor immune response triggers counter-regulatory immunosuppressive mechanisms within the TME [49, 50]. Several lines of evidence support this interpretation. In a murine glioma model, single-cell RNA sequencing revealed that macrophages evolved from an immune-activated phenotype (Stat1-high, Tnf-high) in low-grade tumors to an immunosuppressive state (Apoc1-high, Lgals3-high) in high-grade tumors, suggesting that *APOC1* expression marks a functionally reprogrammed TAM state during malignant progression [51]. Functionally, inhibition of *APOC1* has been shown to reprogram M2-like TAMs toward a pro-inflammatory M1 phenotype via the ferroptosis pathway, thereby enhancing anti-PD-1 efficacy in hepatocellular carcinoma [15] and non-small cell lung cancer [52]. Moreover, *APOC1* expressed in macrophages was recently demonstrated to promote colorectal cancer pulmonary metastasis through the STAT3-CCL2/CCL5 axis, further establishing its role in TAM-mediated tumor promotion [16]. The positive correlations we observed between *APOC1* and immune checkpoint molecules (PD-L1, CTLA-4) are consistent with the compensatory upregulation of inhibitory signals that characterizes adaptive resistance. The finding that *APOC1* is elevated in ICB responders, coupled with its upregulation following IFN-γ and TNF-α stimulation, suggests that *APOC1* may reflect an inflamed TME that, despite harboring immunosuppressive features, retains responsiveness to immunotherapy.

Pharmacogenomic analyses provide initial evidence that *APOC1*-high tumors exhibit distinct therapeutic sensitivity. Association with chemoresistance in BRCA and GBM, coupled with CMap-based identification of MAPK pathway and topoisomerase inhibitors, provides a foundation for biomarker-guided strategies. The consistent negative correlation between *APOC1* expression and MAPK pathway inhibitor sensitivity—indicating resistance—was unexpected given that *APOC1* has been implicated in promoting tumor progression via MAPK signaling activation in colorectal and other cancers [8]. Mechanistically, constitutive ERK activation in APOC1-high tumors may reduce dependence on upstream MEK signaling, conferring resistance akin to adaptive MEK inhibitor resistance observed in BRAF- and KRAS-mutant cancers [53, 54]. Conversely, the identification of the HDAC inhibitor Entinostat (MS-275) as a top compound capable of reversing the *APOC1*-high transcriptomic signature is mechanistically compelling, given our finding that *APOC1* expression correlates with multiple epigenetic regulators, including DNMTs and RNA methylases. Entinostat, a class I HDAC inhibitor, has been shown to reprogram immunosuppressive myeloid cells—including MDSCs and M2-like TAMs—toward a pro-inflammatory, anti-tumor phenotype, and to synergize with immune checkpoint blockade in preclinical models and clinical trials [55, 56]. These observations suggest that the *APOC1*-high TAM state may be sustained by HDAC-dependent epigenetic mechanisms, and that pharmacological disruption of this epigenetic maintenance via Entinostat could represent a rational therapeutic strategy for *APOC1*-high tumors. Together with the cancer type-specific correlations between *APOC1* and DNMT expression observed in our study, these findings support further preclinical evaluation of epigenetic therapies—particularly HDAC inhibition—in combination with immunotherapy for tumors exhibiting an *APOC1*-high, immune-inflamed yet regulated phenotype.

This study has several limitations. First, the observational nature of these analyses precludes causal inferences; experimental studies are needed to establish *APOC1’s* functional contributions. Second, we systematically evaluated the confounding effect of macrophage infiltration on our bulk-level correlations. Partial correlation analyses controlling for M2 abundance confirmed that associations with structural genomic instability (HRD, aneuploidy) were largely independent of immune infiltration, while associations with TMB and MSI were substantially confounded. This analytical framework may inform future TAM-derived biomarker studies. Nonetheless, residual confounding by other cell types cannot be excluded. Third, although validated in multiple independent cohorts, the retrospective analyses introduce potential biases requiring prospective studies with uniform protocols. Finally, our pharmacogenomic predictions—including resistance to MAPK pathway inhibitors and potential sensitivity to Entinostat—are computationally inferred and require experimental validation in *APOC1*-modulated models. Although the MAPK inhibitor resistance finding is consistent across four independent drug sensitivity databases (CTRP, GDSC1, GDSC2, PRISM), and the Entinostat prediction is supported by mechanistic rationale (APOC1-HDAC epigenetic association), these results should be interpreted as hypothesis-generating pending confirmation in patient-derived organoids or xenograft models.

## Conclusion

In summary, this pan-cancer analysis establishes *APOC1* as a context-dependent biomarker with multifaceted roles in tumor biology. After adjusting for macrophage infiltration, we demonstrate that *APOC1*’s associations with structural genomic instability (HRD, aneuploidy) are largely independent of its cellular source, whereas associations with TMB and MSI are substantially confounded by immune infiltration. Rather than reflecting simple immunosuppression, the immunological profile of *APOC1*-high tumors is most consistent with adaptive immune resistance—concurrent immune activation and compensatory inhibition—explaining its elevated expression in immunotherapy responders. From a translational perspective, *APOC1*-high tumors are resistant to MAPK pathway inhibitors but may be vulnerable to epigenetic therapies such as HDAC inhibitors, with the context-dependent prognostic independence of *APOC1* in selected cancers (KIRC, LGG, STAD) further supporting its potential as a biomarker for risk stratification and therapeutic decision-making. These findings provide a foundation for developing *APOC1*-based biomarker strategies and warrant validation in prospective cohorts and experimental models.

## Supplementary Information

Supplementary Material 1. Fig S1. Diagnostic Value of APOC1 Across Cancers. Fig S2. Kaplan-Meier Survival Curves for APOC1 in Representative Cancers. Fig S3. Correlation of APOC1 with Additional Genomic Instability Markers. Fig S4. Correlation Between APOC1 Expression and Epigenetic Regulatory Networks. Fig S5. Correlation Between APOC1 Expression and Tumor Stemness Indices Across Cancers. Fig S6. Clinical Relevance of APOC1 Exon Skip Event APOC1_exon_skip_308128. Fig S7. Protein Interaction Network and GSEA Enrichment Analysis of APOC1. Fig S8. Protein-Level Correlations and APOC1 Dependency Across Cancer Cell Lines. Fig S9. Correlation Between APOC1 Expression and Immune-Related Signatures and Checkpoint Molecules. Fig S10. Comprehensive Characterization of APOC1 in Immune Regulation

Supplementary Material 2. Table S1. Summary of Datasets Used for External Prognostic Validation of APOC1. Table S2. Detailed information of spatial transcriptomic samples analyzed in this study. Table S3. Optimal APOC1 expression cutoffs and permutation-adjusted log-rank test P values for four survival endpoints in 33 cancer types. Table S4. Univariate and multivariate Cox regression analysis of overall survival in cancer types with significant permutation-adjusted log-rank test results. Table S5. Pan-cancer Spearman and partial Spearman correlations between APOC1 expression and genomic instability markers, before and after adjustment for M2 macrophage infiltration. Table S6. Pan-cancer Spearman and partial Spearman correlations between APOC1 expression and stemness indices, before and after adjustment for M2 macrophage infiltration. Table S7: APOC1 clinical-relevant AS events on OncoSplicing. Table S8. GO pathways enriched by the top 100 APOC1 co-expressed genes identified on GEPIA2.0. Table S9: List of abbreviations

## Data Availability

Publicly available datasets were analyzed in this study. RNA sequencing data, somatic mutation data, DNA methylation data, and clinicopathological and survival information for 33 cancer types from The Cancer Genome Atlas (TCGA) and the Genotype-Tissue Expression (GTEx) project were downloaded from the UCSC Xena browser (https://xenabrowser.net/datapages/). Protein expression data were obtained from the Clinical Proteomic Tumor Analysis Consortium (CPTAC) via UALCAN (http://ualcan.path.uab.edu/), and immunohistochemistry images were sourced from the Human Protein Atlas (HPA) database (https://www.proteinatlas.org/). For external prognostic validation, independent cohorts from the Gene Expression Omnibus (GEO), the ArrayExpress database, the Chinese Glioma Genome Atlas (CGGA), the International Cancer Genome Consortium (ICGC), and the DKFZ Heidelberg brain tumor cohort were utilized. A detailed list of these datasets, including accession numbers, cohort identifiers, and platform information, is provided in Supplementary Table S1. Additional analyses utilized the following online repositories: cBioPortal for Cancer Genomics (http://www.cbioportal.org/), the International Cancer Genome Consortium (ICGC) Data Portal (https://dcc.icgc.org/), the DepMap Portal (https://depmap.org/portal/), TISCH (http://tisch.comp-genomics.org/), TISMO (http://tismo.cistrome.org/), ROC Plotter (https://www.rocplotter.com/), the Connectivity Map (CMap) database (https://clue.io/), SpatialTME (https://www.spatialtme.yelab.site/), and SpatialDB (https://www.spatialomics.org/SpatialDB/).
