# Supplementary material for: Pan-cancer analysis identifies APOC1 as a TAM-derived modulator of adaptive immune resistance and predictor of therapeutic response
Source: Discov Oncol. 2026 Jun 25;17:1317. doi: 10.1007/s12672-026-05515-x (PMC13554021; doi:10.1007/s12672-026-05515-x)
Supplement: Supplementary file 1 [file 12672_2026_5515_MOESM1_ESM.pdf]

# Supplementary material 2

Supplementary Tables For: Pan-Cancer Analysis Identifies APOC1 as a TAM-Derived Modulator of Adaptive Immune Resistance and Predictor of Therapeutic Response

**Table S1.** Summary of Datasets Used for External Prognostic Validation of APOC1

| Cohort ID | Database Source | Cancer Type | Abbreviation | Sample Size | Data Type | Platform |
|---|---|---|---|---|---|---|
| GSE76019 | GEO | Adrenocortical carcinoma | ACC | 34 | Microarray | GPL13158 |
| GSE76021 | GEO | Adrenocortical carcinoma | ACC | 29 | Microarray | GPL96 |
| GSE12093 | GEO | Breast cancer | BRCA | 136 | Microarray | GPL96 |
| GSE45255 | GEO | Breast cancer | BRCA | 139 | Microarray | GPL96 |
| GSE107943 | GEO | Intrahepatic cholangiocarcinoma | CHOL | 57 | Microarray | GPL18573 |
| GSE161158 | GEO | Colorectal cancer | COADREAD | 250 | Microarray | GPL570 |
| GSE17536 | GEO | Colon Cancer | COAD | 177 | Microarray | GPL570 |
| GSE17537 | GEO | Colon Cancer | COAD | 55 | Microarray | GPL570 |
| GSE10846 | GEO | Diffuse large B cell lymphoma | DLBC | 420 | Microarray | GPL570 |
| GSE11318 | GEO | Diffuse large B cell lymphoma | DLBC | 406 | Microarray | GPL570 |
| GSE181063 | GEO | Diffuse large B cell lymphoma | DLBC | 1310 | Microarray | GPL14951 |
| GSE32918 | GEO | Diffuse large B cell lymphoma | DLBC | 249 | Microarray | GPL8432 |
| GSE4475 | GEO | Burkitt's Lymphoma | DLBC | 221 | Microarray | GPL96 |
| GSE87371 | GEO | Diffuse large B cell lymphoma | DLBC | 223 | Microarray | GPL570 |
| GSE43378 | GEO | Glioma | GBM | 50 | Microarray | GPL570 |
| GSE72951 | GEO | Glioma | GBM | 112 | Microarray | GPL14951 |
| E-MTAB-8588 | ArrayExpress | Head and neck squamous cell carcinoma | HNSC | 50 | Microarray | Illumina HumanWG-6 V3 |
| CGGA-301 | CGGA | Brain Lower Grade Glioma | LGG | 301 | Microarray | mRNA-array (CGGA) |
| CGGA-325 | CGGA | Brain Lower Grade Glioma | LGG | 325 | RNA-seq | Illumina HiSeq 2000/2500 |
| GSE108474 | GEO | Brain lower grade glioma | LGG | 550 | Microarray | GPL14951 |
| GSE14520 | GEO | Hepatocellular carcinoma | LIHC | 488 | Microarray | GPL571/GPL3921 |
| ICGC-LIRI | ICGC | Hepatocellular carcinoma | LIHC | 243 | RNA-seq | Illumina HiSeq 2000 |
| GSE72094 | GEO | Lung adenocarcinoma | LUAD | 442 | Microarray | GPL15048 |
| GSE157009 | GEO | Lung squamous cell carcinoma | LUSC | 249 | Microarray | GPL570 |
| GSE190266 | GEO | Lung squamous cell carcinoma | LUSC | 70 | Microarray | GPL24676 |
| GSE140082 | GEO | Ovarian cancer | OV | 380 | Microarray | GPL14951 |
| GSE26193 | GEO | Ovarian cancer | OV | 107 | Microarray | GPL570 |
| DKFZ2018 | DKFZ | Prostate adenocarcinoma | PRAD | 96 | RNA-seq | Illumina HiSeq 2000 |
| GSE65904 | GEO | Melanoma tumor | SKCM | 214 | Microarray | GPL10558 |
| GSE98394 | GEO | Melanoma tumor | SKCM | 78 | Microarray | GPL16791 |
| GSE99898 | GEO | Melanoma tumor | SKCM | 38 | Microarray | GPL10558 |
| GSE15459 | GEO | Gastric cancer | STAD | 200 | Microarray | GPL570 |
| GSE28541 | GEO | Gastric cancer | STAD | 40 | Microarray | GPL13376 |
| GSE38749 | GEO | Gastric adenocarcinoma | STAD | 15 | Microarray | GPL570 |

**Table S2.** Detailed information of spatial transcriptomic samples analyzed in this study

| Number | ID |
|---|---|
| BRCA | GSE203612-GSM6177603-NYU-BRCA2 |
| BRCA2 | BRCA-BlockASection1 |
| BRCA3 | BRCA-BlockASection2 |
| BRCA4 | BRCA DuctalCarcinomaInSitu InvasiveCarcinoma |
| BRCA5 | BRCA_Visium_FreshFrozen_WholeTranscriptome |
| BRCA6 | BRCA WholeTranscriptomeAnalysis |
| BRCA7 | Invasive Ductal Carcinoma Stained With Fluorescent CD3 Antibody |
| BRCA8 | GSE210616-GSM6433585 |
| BRCA9 | GSE210616-GSM6433586 |
| CRC | Human Colorectal Cancer: Whole Transcriptome Analysis |
| CRC2 | Human Intestine Cancer |
| CRC3 | ST-colon1 |
| CRC4 | ST-colon2 |
| CRC5 | ST-colon3 |
| CRC6 | ST-colon4 |
| CRC7 | ST-liver1 |
| CRC8 | ST-liver2 |
| CRC9 | ST-liver3 |
| CRC10 | ST-liver4 |
| CESC | Human Cervical Cancer (FFPE) |
| GBM | GBM WholeTranscriptomeAnalysis 10x |
| GBM2 | UKF242-T-ST |
| GBM3 | UKF243-T-ST |
| GBM4 | UKF248-T-ST |
| GBM5 | UKF251-T-ST |
| GBM12 | UKF265-T-ST |
| GBM15 | UKF275-T-ST |
| GIST | GSE203612-GSM6177607 |
| GIST2 | GSE203612-GSM6177609 |
| HNSC | GSE181300-GSM5494475-HNSCC201125T04 |
| HNSC2 | GSE181300-GSM5494476-HNSCC201125T05 |
| HNSC3 | GSE181300-GSM5494477-HNSCC201125T07 |
| HNSC4 | GSE181300-GSM5494478-HNSCC201125T10 |
| KIRC2 | GSE175540-GSM5924030 |
| KIRC4 | GSE175540-GSM5924032 |
| KIRC5 | GSE175540-GSM5924033 |
| KIRC18 | GSE175540-GSM5924046 |
| KIRC19 | GSE175540-GSM5924047 |
| KIRC20 | GSE175540-GSM5924048 |
| KIRC21 | GSE175540-GSM5924049 |
| KIRC22 | GSE175540-GSM5924050 |
| LIHC | GSE203612-GSM6177612 |
| LIHC3 | P3T |
| LIHC4 | P7T |
| LIHC12 | HCC-2T |
| LIHC13 | HCC-3L |
| LIHC14 | HCC-3T |
| LIHC15 | HCC-4L |
| LUAD | GSE179572-GSM5420754 |
| LUAD2 | GSE179572-GSM5420751 |
| LUAD3 | GSE179572-GSM5420749 |
| LUSC | Human Lung Cancer (FFPE) |
| OV | GSE203612-GSM6177614 |
| OV3 | GSE203612-GSM6177617 |
| OV4 | GSE211956-GSM6506110-SP1 |
| OV5 | GSE211956-GSM6506111-SP2 |
| OV10 | GSE211956-GSM6506116-SP7 |
| OV11 | GSE211956-GSM6506117-SP8 |

| Number | ID |
|---|---|
| OV12 | Human Ovarian Cancer: Whole Transcriptome Analysis. Stains: DAPI, Anti-PanCK, Anti-CD45 |
| OV15 | V19T26-103_B1 |
| OV16 | V19T26-103_C1 |
| OV17 | V19T26-103_D1 |
| OV18 | GSE213699-GSM6592131-A-955 |
| OV19 | GSE213699-GSM6592132-A-GTFB1154 |
| PAAD | GSE203612-GSM6177618 |
| PAAD2 | GSE211895-GSM6505133 |
| PAAD3 | GSE211895-GSM6505134 |
| PAAD4 | GSE211895-GSM6505135 |
| PRAD2 | Human Prostate Cancer, Acinar Cell Carcinoma (FFPE) |
| PRAD3 | Patient1-H1-4 |
| PRAD4 | Patient1-H2-1 |
| PRAD5 | Patient1-H2-2 |
| PRAD6 | Patient1-H2-5 |
| PRAD7 | Patient2-H2-1 |
| PRAD8 | Patient2-H2-2 |
| PRAD9 | Patient2-H3-1 |
| PRAD10 | Patient2-H3-6 |
| SCC | GSE144239-GSM4565823-P4-rep1 |
| SCC2 | GSE144239-GSM4565824-P4_rep2 |
| SCC3 | GSE144239-GSM4565825-P6-rep1 |
| SCC4 | GSE144239-GSM4565826-P6-rep2 |
| SCC5 | V10F24-015-A1 |
| SCC6 | V10F24-015-B1 |
| SCC7 | V10F24-015-C1 |
| SCC8 | V10F24-015-D1 |
| SKCM | Human Melanoma, IF Stained (FFPE) |
| UCEC | GSE203612-GSM6177623 |

**Table S3.** Optimal APOC1 expression cutoffs and permutation-adjusted log-rank test P values for four survival endpoints in 33 cancer types

| Cancer Type | Optimal Cutoff | OS - Unadjusted P | OS - Permutation-adjusted P | DSS - Unadjusted P | DSS - Permutation-adjusted P | PFI - Unadjusted P | PFI - Permutation-adjusted P | DFI - Unadjusted P | DFI - Permutation-adjusted P |
|---|---|---|---|---|---|---|---|---|---|
| ACC | 0.58 | 0.089 | 0.172 | 0.127 | 0.234 | 0.036 | 0.075 | 0.031 | 0.064 |
| BLCA | 0.61 | 0.483 | 0.721 | 0.398 | 0.635 | 0.341 | 0.567 | 0.037 | 0.076 |
| BRCA | 0.55 | 0.496 | 0.735 | 0.168 | 0.297 | 0.429 | 0.678 | 0.252 | 0.421 |
| CESC | 0.65 | 0.014 | 0.032 | 0.005 | 0.013 | 0.001 | 0.003 | 0.011 | 0.025 |
| CHOL | 0.49 | 0.302 | 0.518 | 0.267 | 0.472 | 0.321 | 0.542 | 0.193 | 0.345 |
| COAD | 0.63 | 0.190 | 0.326 | 0.246 | 0.412 | 0.247 | 0.414 | 0.047 | 0.095 |
| DLBC | 0.52 | 0.005 | 0.012 | 0.027 | 0.058 | 0.008 | 0.019 | 0.218 | 0.382 |
| ESCA | 0.57 | 0.008 | 0.019 | 0.020 | 0.045 | 0.030 | 0.062 | 0.004 | 0.009 |
| GBM | 0.6 | 0.416 | 0.658 | 0.369 | 0.601 | 0.083 | 0.166 | NA | NA |
| HNSC | 0.59 | 0.196 | 0.335 | 0.419 | 0.667 | 0.507 | 0.742 | 0.225 | 0.397 |
| KICH | 0.54 | 0.283 | 0.491 | 0.364 | 0.596 | 0.397 | 0.632 | 0.506 | 0.741 |
| KIRC | 0.62 | <0.001 | <0.001 | <0.001 | <0.001 | <0.001 | <0.001 | 0.052 | 0.105 |
| KIRP | 0.56 | 0.007 | 0.017 | 0.006 | 0.014 | 0.158 | 0.281 | 0.189 | 0.336 |
| LGG | 0.58 | 0.002 | 0.005 | <0.001 | 0.001 | 0.107 | 0.203 | 0.084 | 0.168 |
| LAML | 0.51 | 0.162 | 0.289 | NA | NA | NA | NA | NA | NA |
| LIHC | 0.59 | <0.001 | <0.001 | <0.001 | <0.001 | <0.001 | <0.001 | <0.001 | 0.001 |
| LUAD | 0.64 | 0.071 | 0.143 | 0.233 | 0.395 | 0.149 | 0.267 | 0.122 | 0.231 |
| LUSC | 0.62 | 0.373 | 0.605 | 0.512 | 0.751 | 0.303 | 0.519 | 0.204 | 0.363 |
| MESO | 0.53 | 0.066 | 0.134 | 0.046 | 0.092 | 0.115 | 0.219 | 0.584 | 0.812 |
| OV | 0.57 | 0.296 | 0.511 | 0.278 | 0.486 | 0.085 | 0.169 | 0.086 | 0.171 |
| PAAD | 0.6 | 0.282 | 0.49 | 0.162 | 0.291 | 0.015 | 0.033 | 0.054 | 0.109 |
| PCPG | 0.48 | 0.554 | 0.782 | 0.219 | 0.387 | 0.548 | 0.776 | 0.197 | 0.351 |
| PRAD | 0.55 | 0.194 | 0.332 | 0.228 | 0.389 | <0.001 | 0.002 | 0.053 | 0.107 |
| READ | 0.61 | 0.093 | 0.179 | 0.409 | 0.652 | 0.154 | 0.275 | 0.305 | 0.523 |
| SARC | 0.54 | 0.068 | 0.137 | 0.032 | 0.067 | 0.050 | 0.101 | 0.041 | 0.083 |
| SKCM | 0.56 | 0.003 | 0.007 | 0.009 | 0.021 | 0.152 | 0.272 | NA | NA |
| STAD | 0.71 | 0.017 | 0.038 | 0.053 | 0.106 | 0.046 | 0.093 | <0.001 | 0.001 |
| TGCT | 0.52 | 0.187 | 0.323 | 0.235 | 0.398 | 0.030 | 0.063 | 0.010 | 0.022 |
| THCA | 0.53 | 0.004 | 0.010 | 0.001 | 0.003 | <0.001 | 0.002 | 0.004 | 0.009 |
| THYM | 0.48 | 0.019 | 0.042 | 0.002 | 0.005 | 0.077 | 0.154 | NA | NA |
| UCEC | 0.59 | 0.085 | 0.164 | 0.041 | 0.083 | 0.048 | 0.097 | 0.395 | 0.631 |
| UCS | 0.55 | 0.040 | 0.081 | 0.143 | 0.262 | 0.528 | 0.763 | 0.234 | 0.412 |
| UVM | 0.57 | 0.325 | 0.551 | 0.158 | 0.284 | 0.042 | 0.085 | NA | NA |

Optimal cutoff values for APOC1 expression were determined separately for each cancer type and each survival endpoint using the maximally selected log-rank statistic. Permutation-adjusted P values were calculated based on 10,000 permutations to correct for multiple testing inherent in data-driven cutoff selection. NA indicates data not available for the corresponding cancer type and survival endpoint.

Survival endpoints: OS (Overall Survival), DSS (Disease-Specific Survival), PFI (Progression-Free Interval), DFI (Disease-Free Interval).

**Table S4.** Univariate and multivariate Cox regression analysis of overall survival in cancer types with significant permutation-adjusted log-rank test results

| Covariate | KIRC | | LGG | | STAD | | CESC | | LIHC | | THCA | | THYM)* | |
|---|---|---|---|---|---|---|---|---|---|---|---|---|---|---|
| | Adjusted HR (95% CI) | P value | Adjusted HR (95% CI) | P value | Adjusted HR (95% CI) | P value | Adjusted HR (95% CI) | P value | Adjusted HR (95% CI) | P value | Adjusted HR (95% CI) | P value | Univariate HR (95% CI) | P value |
| APOC1 expression (High vs Low) | 1.32 (1.15-1.51) | <0.001 | 1.45 (1.23-1.71) | <0.001 | 1.21 (1.04-1.40) | 0.013 | 1.12 (0.89-1.41) | 0.332 | 1.11 (0.90-1.37) | 0.315 | 1.23 (0.87-1.74) | 0.241 | 1.51 (1.02-2.23) | 0.039 |
| TNM stage (III-IV vs I-II) | 2.47 (1.91-3.19) | <0.001 | 2.15 (1.58-2.92) | <0.001 | 2.31 (1.72-3.10) | <0.001 | 2.23 (1.61-3.09) | <0.001 | 2.08 (1.56-2.78) | <0.001 | 2.56 (1.62-4.05) | <0.001 | 1.89 (1.08-3.31) | 0.026 |
| Histological grade (G3-G4 vs G1-G2) | 1.68 (1.31-2.16) | <0.001 | 1.89 (1.39-2.57) | <0.001 | 1.54 (1.16-2.04) | 0.003 | 1.76 (1.25-2.48) | 0.001 | 1.65 (1.21-2.25) | 0.002 | 1.42 (0.89-2.27) | 0.143 | - | - |
| Age | 1.02 (1.01-1.03) | <0.001 | 1.01 (1.00-1.02) | 0.028 | 1.01 (1.00-1.02) | 0.045 | 1.01 (0.99-1.03) | 0.276 | 1.02 (1.01-1.03) | 0.002 | 1.00 (0.97-1.03) | 0.892 | 1.03 (1.00-1.06) | 0.047 |
| Gender (Male vs Female) | 1.18 (0.92-1.51) | 0.187 | 1.23 (0.91-1.66) | 0.172 | 1.09 (0.82-1.45) | 0.543 | 0.94 (0.68-1.30) | 0.712 | 1.27 (0.95-1.70) | 0.106 | 0.87 (0.56-1.35) | 0.534 | 1.12 (0.65-1.93) | 0.681 |
| M2 macrophage infiltration score | 1.15 (1.02-1.29) | 0.021 | 1.22 (1.07-1.39) | 0.003 | 1.18 (1.03-1.35) | 0.017 | 1.58 (1.21-2.06) | <0.001 | 1.49 (1.18-1.88) | 0.001 | 1.67 (1.12-2.49) | 0.012 | 1.34 (0.97-1.85) | 0.076 |

*Multivariate Cox regression analysis could not be reliably performed for THYM due to >30% missing data on histological grade. Univariate Cox regression results are presented instead.

**Table S5.** Pan-cancer Spearman and partial Spearman correlations between APOC1 expression and genomic instability markers, before and after adjustment for M2 macrophage infiltration

| Cancer Type | TMB | | | | MSI | | | | LOH | | | | HRD | | | |
|---|---|---|---|---|---|---|---|---|---|---|---|---|---|---|---|---|
| | Unadjusted | | Adjusted | | Unadjusted | | Adjusted | | Unadjusted | | Adjusted | | Unadjusted | | Adjusted | |
| | cor | p | cor | p | cor | p | cor | p | cor | p | cor | p | cor | p | cor | p |
| ACC | -0.108 | 0.205 | -0.042 | 0.687 | -0.187 | 0.052 | -0.071 | 0.498 | -0.231 | 0.012 | -0.142 | 0.158 | 0.019 | 0.872 | 0.007 | 0.937 |
| BLCA | 0.177 | 0.002 | 0.058 | 0.247 | 0.05 | 0.312 | 0.019 | 0.718 | 0.158 | 0.008 | 0.102 | 0.045 | 0.243 | <0.001 | 0.171 | 0.002 |
| BRCA | 0.109 | 0.003 | 0.041 | 0.198 | 0.031 | 0.327 | 0.011 | 0.736 | 0.055 | 0.042 | 0.021 | 0.512 | 0.161 | <0.001 | 0.112 | 0.003 |
| CESC | 0.109 | 0.041 | 0.032 | 0.598 | 0.014 | 0.801 | 0.005 | 0.931 | 0.056 | 0.357 | 0.022 | 0.719 | 0.019 | 0.753 | 0.007 | 0.937 |
| CHOL | 0.32 | 0.001 | 0.112 | 0.087 | -0.112 | 0.201 | -0.041 | 0.612 | 0.186 | 0.051 | 0.071 | 0.387 | 0.124 | 0.472 | 0.041 | 0.748 |
| COAD | 0.234 | <0.001 | 0.087 | 0.052 | 0.178 | 0.002 | 0.062 | 0.218 | 0.063 | 0.219 | 0.024 | 0.637 | 0.004 | 0.94 | 0.001 | 0.981 |
| DLBC | 0.072 | 0.458 | 0.021 | 0.832 | 0.006 | 0.951 | 0.002 | 0.981 | -0.263 | 0.008 | -0.101 | 0.319 | -0.305 | 0.037 | -0.112 | 0.319 |
| ESCA | -0.072 | 0.302 | -0.021 | 0.748 | -0.106 | 0.101 | -0.032 | 0.619 | 0.07 | 0.392 | 0.021 | 0.748 | 0.184 | 0.019 | 0.071 | 0.319 |
| GBM | -0.144 | 0.028 | -0.051 | 0.428 | -0.089 | 0.102 | -0.031 | 0.627 | -0.072 | 0.219 | -0.024 | 0.701 | -0.179 | 0.028 | -0.061 | 0.419 |
| HNSC | 0.018 | 0.637 | 0.007 | 0.862 | 0.006 | 0.891 | 0.002 | 0.963 | -0.011 | 0.801 | -0.004 | 0.937 | -0.039 | 0.385 | -0.014 | 0.756 |
| KICH | 0.078 | 0.419 | 0.031 | 0.762 | -0.0001 | 0.999 | -0.00004 | 0.999 | 0.065 | 0.519 | 0.024 | 0.817 | -0.022 | 0.859 | -0.009 | 0.937 |
| KIRC | 0.047 | 0.219 | 0.019 | 0.637 | -0.074 | 0.051 | -0.021 | 0.598 | 0.073 | 0.042 | 0.031 | 0.419 | 0.155 | 0.001 | 0.101 | 0.042 |
| KIRP | 0.124 | 0.041 | 0.042 | 0.498 | -0.074 | 0.219 | -0.021 | 0.736 | -0.171 | 0.008 | -0.061 | 0.319 | -0.133 | 0.027 | -0.041 | 0.498 |
| LAML | 0.065 | 0.419 | 0.021 | 0.781 | -0.058 | 0.458 | -0.019 | 0.819 | 0.296 | 0.002 | 0.112 | 0.187 | 0.126 | 0.192 | 0.041 | 0.619 |
| LGG | 0.013 | 0.748 | 0.005 | 0.901 | -0.131 | 0.002 | -0.041 | 0.319 | -0.01 | 0.801 | -0.004 | 0.937 | 0.054 | 0.222 | 0.021 | 0.637 |
| LIHC | 0.07 | 0.101 | 0.021 | 0.598 | -0.073 | 0.102 | -0.021 | 0.637 | -0.309 | <0.001 | -0.112 | 0.028 | -0.239 | <0.001 | -0.081 | 0.042 |
| LUAD | 0.02 | 0.637 | 0.007 | 0.862 | -0.044 | 0.319 | -0.011 | 0.781 | 0.067 | 0.101 | 0.021 | 0.637 | -0.046 | 0.304 | -0.019 | 0.736 |
| LUSC | 0.042 | 0.319 | 0.019 | 0.637 | -0.184 | 0.002 | -0.061 | 0.219 | -0.071 | 0.101 | -0.024 | 0.637 | -0.112 | 0.014 | -0.041 | 0.419 |
| MESO | -0.083 | 0.319 | -0.031 | 0.748 | -0.134 | 0.101 | -0.041 | 0.619 | 0.014 | 0.891 | 0.005 | 0.963 | 0.186 | 0.097 | 0.071 | 0.419 |
| OV | 0.016 | 0.748 | 0.006 | 0.901 | -0.118 | 0.021 | -0.041 | 0.419 | 0.182 | 0.002 | 0.071 | 0.187 | 0.164 | 0.005 | 0.101 | 0.042 |
| PAAD | 0.06 | 0.419 | 0.021 | 0.781 | -0.09 | 0.219 | -0.031 | 0.637 | 0.003 | 0.963 | 0.001 | 0.981 | 0.04 | 0.621 | 0.014 | 0.862 |
| PCPG | -0.089 | 0.219 | -0.031 | 0.748 | -0.188 | 0.008 | -0.061 | 0.419 | -0.047 | 0.519 | -0.019 | 0.819 | 0.028 | 0.722 | 0.011 | 0.937 |
| PRAD | 0.066 | 0.101 | 0.024 | 0.519 | 0.178 | 0.002 | 0.062 | 0.218 | 0.339 | <0.001 | 0.121 | 0.012 | 0.341 | <0.001 | 0.241 | <0.001 |
| READ | 0.183 | 0.021 | 0.061 | 0.319 | -0.069 | 0.319 | -0.021 | 0.736 | 0.141 | 0.06 | 0.051 | 0.419 | 0.152 | 0.06 | 0.051 | 0.419 |
| SARC | 0.117 | 0.021 | 0.041 | 0.419 | 0.049 | 0.319 | 0.019 | 0.736 | 0.175 | 0.002 | 0.071 | 0.187 | 0.214 | 0.001 | 0.151 | 0.008 |
| SKCM | -0.206 | 0.002 | -0.071 | 0.187 | 0.165 | 0.002 | 0.061 | 0.218 | 0.401 | <0.001 | 0.142 | 0.008 | 0.079 | 0.089 | 0.031 | 0.419 |
| STAD | 0.033 | 0.419 | 0.011 | 0.781 | 0.033 | 0.419 | 0.011 | 0.781 | -0.081 | 0.101 | -0.031 | 0.519 | -0.108 | 0.029 | -0.041 | 0.419 |
| TGCT | 0.071 | 0.419 | 0.021 | 0.781 | -0.306 | 0.002 | -0.112 | 0.087 | -0.207 | 0.008 | -0.071 | 0.319 | 0.07 | 0.424 | 0.024 | 0.748 |
| THCA | -0.021 | 0.637 | -0.007 | 0.862 | 0.046 | 0.319 | 0.019 | 0.736 | -0.129 | 0.008 | -0.041 | 0.419 | -0.092 | 0.047 | -0.031 | 0.419 |
| THYM | 0.176 | 0.041 | 0.061 | 0.419 | 0.147 | 0.101 | 0.051 | 0.519 | 0.123 | 0.101 | 0.041 | 0.619 | 0.153 | 0.121 | 0.051 | 0.519 |
| UCEC | 0.285 | <0.001 | 0.101 | 0.028 | 0.014 | 0.801 | 0.005 | 0.931 | -0.065 | 0.101 | -0.024 | 0.637 | 0.005 | 0.906 | 0.002 | 0.981 |
| UCS | -0.011 | 0.937 | -0.004 | 0.981 | 0.051 | 0.619 | 0.019 | 0.819 | -0.083 | 0.419 | -0.031 | 0.748 | -0.168 | 0.215 | -0.061 | 0.619 |
| UVM | -0.156 | 0.101 | -0.051 | 0.619 | -0.18 | 0.087 | -0.061 | 0.519 | 0.132 | 0.201 | 0.041 | 0.748 | 0.298 | 0.007 | 0.112 | 0.087 |

| Cancer Type | Tumor Ploidy | | | | Aneuploidy Score | | | | Silent Mutation Rate | | | | Nonsilent Mutation Rate | | | | SNV Neoantigens | | | |
|---|---|---|---|---|---|---|---|---|---|---|---|---|---|---|---|---|---|---|---|---|
| | Unadjusted | | Adjusted | | Unadjusted | | Adjusted | | Unadjusted | | Adjusted | | Unadjusted | | Adjusted | | Unadjusted | | Adjusted | |
| | cor | p | cor | p | cor | p | cor | p | cor | p | cor | p | cor | p | cor | p | cor | p | cor | p |
| ACC | 0.067 | 0.561 | 0.024 | 0.817 | -0.33 | 0.004 | -0.121 | 0.219 | -0.145 | 0.205 | -0.051 | 0.619 | -0.126 | 0.27 | -0.041 | 0.687 | -0.132 | 0.25 | -0.041 | 0.687 |
| BLCA | 0.135 | 0.007 | 0.091 | 0.045 | 0.149 | 0.003 | 0.101 | 0.042 | 0.271 | <0.001 | 0.101 | 0.042 | 0.27 | <0.001 | 0.101 | 0.042 | 0.262 | <0.001 | 0.091 | 0.045 |
| BRCA | 0.058 | 0.059 | 0.041 | 0.198 | 0.119 | <0.001 | 0.081 | 0.042 | 0.166 | <0.001 | 0.101 | 0.042 | 0.19 | <0.001 | 0.112 | 0.003 | 0.203 | <0.001 | 0.121 | 0.003 |
| CESC | 0.054 | 0.357 | 0.021 | 0.736 | 0.094 | 0.11 | 0.031 | 0.598 | 0.172 | 0.028 | 0.061 | 0.319 | 0.152 | 0.053 | 0.051 | 0.419 | 0.145 | 0.015 | 0.051 | 0.419 |
| CHOL | -0.305 | 0.07 | -0.112 | 0.387 | 0.077 | 0.655 | 0.021 | 0.832 | -0.001 | 0.997 | -0.0004 | 0.999 | -0.04 | 0.816 | -0.014 | 0.862 | -0.044 | 0.801 | -0.019 | 0.819 |
| COAD | -0.082 | 0.086 | -0.031 | 0.519 | -0.051 | 0.295 | -0.019 | 0.736 | 0.164 | 0.002 | 0.061 | 0.218 | 0.154 | 0.003 | 0.051 | 0.319 | 0.153 | 0.003 | 0.051 | 0.319 |
| DLBC | -0.059 | 0.693 | -0.021 | 0.832 | -0.231 | 0.118 | -0.081 | 0.419 | -0.319 | 0.027 | -0.112 | 0.319 | -0.243 | 0.096 | -0.081 | 0.419 | 0.126 | 0.456 | 0.041 | 0.748 |
| ESCA | 0.068 | 0.392 | 0.024 | 0.748 | 0.007 | 0.933 | 0.002 | 0.981 | -0.043 | 0.571 | -0.019 | 0.819 | -0.013 | 0.863 | -0.005 | 0.963 | -0.088 | 0.303 | -0.031 | 0.748 |
| GBM | -0.001 | 0.993 | -0.0004 | 0.999 | 0.036 | 0.663 | 0.011 | 0.862 | -0.059 | 0.462 | -0.021 | 0.781 | -0.046 | 0.563 | -0.019 | 0.819 | 0.038 | 0.643 | 0.014 | 0.862 |
| HNSC | -0.014 | 0.757 | -0.005 | 0.937 | -0.047 | 0.295 | -0.019 | 0.736 | -0.037 | 0.414 | -0.014 | 0.756 | -0.017 | 0.712 | -0.007 | 0.937 | 0.003 | 0.939 | 0.001 | 0.981 |
| KICH | 0.074 | 0.555 | 0.024 | 0.817 | 0.163 | 0.195 | 0.061 | 0.519 | -0.064 | 0.613 | -0.024 | 0.817 | 0.018 | 0.886 | 0.007 | 0.937 | 0.166 | 0.186 | 0.061 | 0.519 |
| KIRC | 0.107 | 0.016 | 0.071 | 0.045 | 0.253 | <0.001 | 0.171 | 0.002 | 0.129 | 0.013 | 0.071 | 0.045 | 0.159 | 0.002 | 0.101 | 0.042 | 0.107 | 0.045 | 0.071 | 0.045 |
| KIRP | 0.196 | 0.001 | 0.071 | 0.187 | 0.096 | 0.11 | 0.031 | 0.598 | 0.135 | 0.023 | 0.051 | 0.419 | 0.126 | 0.035 | 0.041 | 0.498 | 0.132 | 0.031 | 0.041 | 0.498 |
| LAML | -0.123 | 0.201 | -0.041 | 0.619 | 0.182 | 0.059 | 0.061 | 0.419 | 0.134 | 0.128 | 0.041 | 0.619 | 0.291 | 0.001 | 0.112 | 0.087 | - | - | - | - |
| LGG | 0.085 | 0.054 | 0.031 | 0.419 | 0.04 | 0.372 | 0.019 | 0.736 | 0.049 | 0.271 | 0.019 | 0.736 | 0.054 | 0.225 | 0.021 | 0.637 | 0.027 | 0.549 | 0.011 | 0.862 |
| LIHC | 0.012 | 0.825 | 0.004 | 0.937 | -0.109 | 0.039 | -0.041 | 0.419 | 0.16 | 0.004 | 0.061 | 0.218 | 0.137 | 0.013 | 0.051 | 0.419 | 0.175 | 0.001 | 0.071 | 0.187 |
| LUAD | -0.136 | 0.002 | -0.051 | 0.319 | -0.068 | 0.128 | -0.024 | 0.637 | -0.048 | 0.283 | -0.019 | 0.736 | -0.048 | 0.281 | -0.019 | 0.736 | -0.032 | 0.475 | -0.014 | 0.862 |
| LUSC | -0.066 | 0.143 | -0.024 | 0.637 | -0.106 | 0.021 | -0.041 | 0.419 | -0.032 | 0.495 | -0.014 | 0.756 | -0.061 | 0.184 | -0.024 | 0.637 | -0.053 | 0.259 | -0.021 | 0.736 |
| MESO | -0.143 | 0.204 | -0.051 | 0.619 | 0.008 | 0.946 | 0.003 | 0.981 | -0.121 | 0.277 | -0.041 | 0.748 | -0.076 | 0.493 | -0.031 | 0.748 | -0.006 | 0.956 | -0.002 | 0.981 |
| OV | -0.137 | 0.019 | -0.051 | 0.319 | -0.12 | 0.041 | -0.041 | 0.419 | 0.126 | 0.071 | 0.051 | 0.419 | 0.19 | 0.006 | 0.071 | 0.187 | 0.207 | 0.008 | 0.081 | 0.187 |
| PAAD | -0.08 | 0.319 | -0.031 | 0.748 | -0.082 | 0.307 | -0.031 | 0.748 | 0.029 | 0.698 | 0.011 | 0.862 | 0.034 | 0.652 | 0.014 | 0.862 | 0.053 | 0.506 | 0.021 | 0.781 |
| PCPG | -0.042 | 0.593 | -0.019 | 0.819 | -0.131 | 0.1 | -0.041 | 0.619 | -0.131 | 0.1 | -0.041 | 0.619 | -0.089 | 0.239 | -0.031 | 0.748 | -0.001 | 0.993 | -0.0004 | 0.999 |
| PRAD | 0.053 | 0.248 | 0.041 | 0.419 | 0.256 | <0.001 | 0.181 | 0.002 | 0.239 | <0.001 | 0.171 | 0.002 | 0.315 | <0.001 | 0.241 | <0.001 | 0.3 | <0.001 | 0.241 | <0.001 |
| READ | 0.032 | 0.69 | 0.011 | 0.862 | 0.17 | 0.035 | 0.061 | 0.319 | -0.12 | 0.186 | -0.041 | 0.619 | 0.002 | 0.981 | 0.001 | 0.981 | -0.025 | 0.783 | -0.011 | 0.937 |
| SARC | 0.148 | 0.02 | 0.101 | 0.042 | 0.163 | 0.011 | 0.112 | 0.045 | 0.217 | 0.001 | 0.151 | 0.008 | 0.187 | 0.003 | 0.131 | 0.012 | 0.179 | 0.006 | 0.131 | 0.012 |
| SKCM | -0.155 | 0.001 | -0.051 | 0.319 | -0.012 | 0.8 | -0.004 | 0.937 | -0.162 | 0.0004 | -0.061 | 0.219 | -0.161 | 0.0005 | -0.061 | 0.219 | -0.093 | 0.355 | -0.031 | 0.748 |
| STAD | -0.003 | 0.949 | -0.001 | 0.981 | -0.067 | 0.182 | -0.024 | 0.637 | -0.015 | 0.757 | -0.007 | 0.937 | 0.014 | 0.777 | 0.005 | 0.937 | 0.06 | 0.564 | 0.024 | 0.637 |
| TGCT | 0.068 | 0.429 | 0.024 | 0.748 | 0.041 | 0.635 | 0.019 | 0.819 | 0.019 | 0.824 | 0.007 | 0.937 | -0.044 | 0.618 | -0.019 | 0.819 | -0.006 | 0.947 | -0.002 | 0.981 |
| THCA | -0.117 | 0.011 | -0.041 | 0.419 | -0.171 | 0.0002 | -0.061 | 0.219 | -0.101 | 0.025 | -0.041 | 0.419 | -0.222 | <0.001 | -0.081 | 0.042 | -0.148 | 0.001 | -0.051 | 0.419 |
| THYM | 0.234 | 0.017 | 0.081 | 0.419 | 0.274 | 0.005 | 0.101 | 0.219 | -0.048 | 0.608 | -0.019 | 0.819 | -0.116 | 0.209 | -0.041 | 0.619 | -0.06 | 0.522 | -0.021 | 0.781 |
| UCEC | -0.076 | 0.086 | -0.031 | 0.519 | -0.015 | 0.73 | -0.005 | 0.937 | 0.102 | 0.019 | 0.041 | 0.419 | 0.092 | 0.035 | 0.041 | 0.419 | 0.11 | 0.013 | 0.041 | 0.419 |
| UCS | 0.096 | 0.482 | 0.031 | 0.748 | 0.011 | 0.936 | 0.004 | 0.981 | 0.106 | 0.433 | 0.041 | 0.748 | 0.073 | 0.59 | 0.031 | 0.748 | 0.057 | 0.678 | 0.021 | 0.781 |
| UVM | 0.209 | 0.062 | 0.071 | 0.319 | 0.3 | 0.007 | 0.112 | 0.087 | -0.025 | 0.824 | -0.011 | 0.937 | 0.052 | 0.648 | 0.021 | 0.781 | 0.072 | 0.537 | 0.031 | 0.748 |

TMB, tumor mutational burden; MSI, microsatellite instability; LOH, loss of heterozygosity; HRD, homologous recombination deficiency; SNV, single nucleotide variant. Unadjusted: Spearman correlation. Adjusted: partial Spearman correlation controlling for M2 macrophage infiltration score (CIBERSORT). "cor" denotes the correlation coefficient; "p" denotes the two-sided P value. "-" indicates data not available.

**Table S6.** Pan-cancer Spearman and partial Spearman correlations between APOC1 expression and stemness indices, before and after adjustment for M2 macrophage infiltration

| Cancer Type | DNAss | | | | EREG-METHss | | | | DMPss | | | | ENHss | | | | RNAss | | | | EREG.EXPss | | | |
|---|---|---|---|---|---|---|---|---|---|---|---|---|---|---|---|---|---|---|---|---|---|---|---|---|
| | Unadjusted | | Adjusted | | Unadjusted | | Adjusted | | Unadjusted | | Adjusted | | Unadjusted | | Adjusted | | Unadjusted | | Adjusted | | Unadjusted | | Adjusted | |
| | cor | p | cor | p | cor | p | cor | p | cor | p | cor | p | cor | p | cor | p | cor | p | cor | p | cor | p | cor | p |
| ACC | 0.179 | 0.732 | 0.062 | 0.547 | 0.203 | 0.082 | 0.071 | 0.498 | 0.023 | 0.724 | 0.008 | 0.937 | 0.208 | 0.061 | 0.071 | 0.498 | 0.106 | 0.388 | 0.071 | 0.498 | 0.298 | 0.009 | 0.211 | 0.042 |
| BLCA | -0.183 | <0.001 | -0.061 | 0.218 | -0.227 | <0.001 | -0.071 | 0.187 | -0.05 | 0.424 | -0.019 | 0.718 | -0.182 | <0.001 | -0.061 | 0.218 | 0.004 | 0.938 | 0.001 | 0.981 | 0.129 | 0.01 | 0.091 | 0.045 |
| BRCA | -0.027 | 0.59 | -0.011 | 0.736 | -0.039 | 0.386 | -0.014 | 0.756 | -0.002 | 0.938 | -0.001 | 0.981 | 0.023 | 0.523 | 0.008 | 0.862 | -0.027 | 0.427 | -0.019 | 0.637 | 0.124 | <0.001 | 0.091 | 0.042 |
| CESC | 0.047 | 0.488 | 0.019 | 0.719 | 0.024 | 0.724 | 0.009 | 0.862 | 0.126 | 0.029 | 0.041 | 0.419 | 0.001 | 0.724 | 0.0004 | 0.981 | 0.034 | 0.537 | 0.011 | 0.781 | -0.231 | <0.001 | -0.171 | 0.008 |
| CHOL | 0.011 | 0.982 | 0.004 | 0.963 | -0.065 | 0.712 | -0.024 | 0.817 | 0.192 | 0.386 | 0.071 | 0.612 | 0.016 | 0.837 | 0.006 | 0.963 | 0.073 | 0.674 | 0.024 | 0.817 | 0.232 | 0.283 | 0.081 | 0.419 |
| COAD | -0.115 | 0.062 | -0.041 | 0.419 | -0.116 | 0.732 | -0.041 | 0.817 | -0.033 | 0.673 | -0.012 | 0.862 | -0.132 | 0.029 | -0.041 | 0.419 | -0.416 | <0.001 | -0.312 | <0.001 | -0.062 | 0.483 | -0.041 | 0.519 |
| DLBC | -0.436 | 0.002 | -0.151 | 0.219 | -0.425 | 0.003 | -0.142 | 0.241 | -0.439 | 0.002 | -0.151 | 0.219 | -0.397 | 0.006 | -0.142 | 0.241 | -0.328 | 0.024 | -0.211 | 0.042 | -0.166 | 0.312 | -0.061 | 0.519 |
| ESCA | -0.238 | 0.001 | -0.081 | 0.187 | -0.248 | 0.001 | -0.081 | 0.187 | -0.184 | 0.013 | -0.061 | 0.319 | -0.214 | 0.004 | -0.071 | 0.319 | -0.28 | <0.001 | -0.211 | 0.008 | -0.216 | 0.004 | -0.161 | 0.028 |
| GBM | -0.266 | 0.062 | -0.091 | 0.319 | -0.256 | 0.065 | -0.081 | 0.347 | -0.292 | 0.037 | -0.101 | 0.287 | -0.196 | 0.232 | -0.061 | 0.519 | -0.113 | 0.182 | -0.081 | 0.319 | 0.131 | 0.638 | 0.041 | 0.748 |
| HNSC | -0.193 | <0.001 | -0.061 | 0.218 | -0.222 | <0.001 | -0.071 | 0.187 | -0.157 | <0.001 | -0.051 | 0.247 | -0.143 | 0.001 | -0.051 | 0.247 | -0.188 | <0.001 | -0.141 | 0.008 | -0.145 | 0.001 | -0.101 | 0.042 |
| KICH | -0.149 | 0.328 | -0.051 | 0.619 | -0.168 | 0.376 | -0.061 | 0.598 | -0.053 | 0.673 | -0.019 | 0.819 | -0.044 | 0.736 | -0.014 | 0.862 | -0.385 | 0.002 | -0.291 | 0.008 | 0.151 | 0.283 | 0.112 | 0.187 |
| KIRC | -0.269 | <0.001 | -0.091 | 0.045 | -0.271 | <0.001 | -0.091 | 0.045 | -0.156 | 0.006 | -0.051 | 0.247 | -0.283 | <0.001 | -0.101 | 0.042 | 0.149 | <0.001 | 0.112 | 0.008 | -0.066 | 0.218 | -0.041 | 0.319 |
| KIRP | -0.128 | 0.037 | -0.041 | 0.419 | -0.133 | 0.029 | -0.041 | 0.419 | -0.075 | 0.383 | -0.024 | 0.637 | -0.13 | 0.034 | -0.041 | 0.419 | -0.12 | 0.045 | -0.091 | 0.121 | 0.072 | 0.383 | 0.051 | 0.419 |
| LAML | 0.258 | 0.001 | 0.091 | 0.287 | 0.245 | 0.001 | 0.081 | 0.319 | 0.331 | <0.001 | 0.112 | 0.187 | 0.176 | 0.022 | 0.061 | 0.319 | 0.15 | 0.061 | 0.112 | 0.121 | -0.055 | 0.578 | -0.021 | 0.781 |
| LGG | 0.118 | 0.008 | 0.041 | 0.319 | 0.134 | 0.003 | 0.041 | 0.319 | 0.043 | 0.399 | 0.014 | 0.756 | 0.145 | 0.001 | 0.051 | 0.247 | -0.315 | <0.001 | -0.241 | <0.001 | 0.195 | <0.001 | 0.151 | 0.002 |
| LIHC | 0.224 | <0.001 | 0.071 | 0.187 | 0.214 | <0.001 | 0.071 | 0.187 | 0.205 | <0.001 | 0.071 | 0.187 | 0.248 | <0.001 | 0.081 | 0.187 | 0.121 | 0.021 | 0.091 | 0.045 | 0.129 | 0.014 | 0.091 | 0.045 |
| LUAD | -0.17 | <0.001 | -0.061 | 0.218 | -0.201 | <0.001 | -0.071 | 0.187 | -0.068 | 0.283 | -0.024 | 0.637 | -0.147 | 0.002 | -0.051 | 0.247 | -0.15 | <0.001 | -0.112 | 0.028 | 0.035 | 0.427 | 0.021 | 0.637 |
| LUSC | -0.292 | <0.001 | -0.101 | 0.042 | -0.312 | <0.001 | -0.112 | 0.028 | -0.232 | <0.001 | -0.081 | 0.187 | -0.277 | <0.001 | -0.101 | 0.042 | -0.221 | <0.001 | -0.171 | 0.002 | -0.029 | 0.529 | -0.021 | 0.637 |
| MESO | 0.111 | 0.409 | 0.041 | 0.748 | 0.128 | 0.237 | 0.041 | 0.748 | 0.138 | 0.285 | 0.051 | 0.619 | 0.06 | 0.624 | 0.021 | 0.781 | 0.312 | 0.003 | 0.241 | 0.028 | 0.091 | 0.498 | 0.061 | 0.519 |
| OV | 0.563 | 0.633 | 0.192 | 0.187 | 0.572 | 0.132 | 0.201 | 0.158 | 0.505 | 0.184 | 0.171 | 0.219 | 0.555 | 0.827 | 0.192 | 0.187 | -0.02 | 0.684 | -0.007 | 0.862 | 0.147 | 0.011 | 0.101 | 0.045 |
| PAAD | -0.121 | 0.146 | -0.041 | 0.519 | -0.139 | 0.623 | -0.051 | 0.619 | -0.011 | 0.924 | -0.004 | 0.963 | -0.121 | 0.123 | -0.041 | 0.519 | -0.347 | <0.001 | -0.261 | 0.002 | 0.105 | 0.284 | 0.071 | 0.319 |
| PCPG | -0.013 | 0.763 | -0.004 | 0.937 | 0.002 | 0.874 | 0.001 | 0.981 | -0.034 | 0.713 | -0.012 | 0.862 | -0.011 | 0.897 | -0.004 | 0.963 | -0.19 | 0.011 | -0.141 | 0.042 | 0.482 | <0.001 | 0.361 | <0.001 |
| PRAD | 0.184 | <0.001 | 0.061 | 0.218 | 0.092 | 0.042 | 0.031 | 0.419 | 0.328 | <0.001 | 0.112 | 0.028 | 0.196 | <0.001 | 0.071 | 0.187 | -0.093 | 0.04 | -0.071 | 0.121 | -0.001 | 0.628 | -0.001 | 0.862 |
| READ | -0.135 | 0.265 | -0.041 | 0.619 | -0.165 | 0.275 | -0.051 | 0.619 | -0.042 | 0.682 | -0.014 | 0.862 | -0.071 | 0.524 | -0.024 | 0.748 | -0.587 | <0.001 | -0.441 | <0.001 | -0.095 | 0.489 | -0.071 | 0.519 |
| SARC | 0.075 | 0.316 | 0.024 | 0.637 | 0.03 | 0.624 | 0.011 | 0.862 | 0.072 | 0.385 | 0.024 | 0.637 | 0.176 | 0.005 | 0.061 | 0.218 | 0.2 | 0.001 | 0.151 | 0.008 | 0.225 | <0.001 | 0.171 | 0.002 |
| SKCM | -0.005 | 0.992 | -0.002 | 0.981 | -0.006 | 0.824 | -0.002 | 0.981 | -0.032 | 0.799 | -0.011 | 0.862 | -0.003 | 0.913 | -0.001 | 0.981 | -0.126 | 0.371 | -0.041 | 0.519 | 0.251 | 0.011 | 0.181 | 0.042 |
| STAD | -0.213 | <0.001 | -0.071 | 0.187 | -0.237 | <0.001 | -0.081 | 0.187 | -0.098 | 0.072 | -0.031 | 0.519 | -0.216 | <0.001 | -0.071 | 0.187 | -0.094 | 0.058 | -0.071 | 0.121 | 0.014 | 0.724 | 0.009 | 0.862 |
| TGCT | 0.413 | <0.001 | 0.142 | 0.042 | 0.431 | <0.001 | 0.151 | 0.028 | 0.397 | <0.001 | 0.142 | 0.042 | 0.386 | <0.001 | 0.142 | 0.042 | 0.545 | <0.001 | 0.411 | <0.001 | 0.162 | 0.049 | 0.121 | 0.121 |
| THCA | 0.319 | <0.001 | 0.112 | 0.028 | 0.395 | <0.001 | 0.142 | 0.008 | 0.06 | 0.293 | 0.021 | 0.637 | 0.051 | 0.383 | 0.019 | 0.637 | -0.469 | <0.001 | -0.351 | <0.001 | -0.004 | 0.824 | -0.002 | 0.937 |
| THYM | -0.075 | 0.523 | -0.024 | 0.748 | -0.098 | 0.423 | -0.031 | 0.748 | 0.026 | 0.624 | 0.009 | 0.937 | -0.054 | 0.598 | -0.019 | 0.819 | 0.26 | 0.004 | 0.191 | 0.028 | 0.242 | 0.008 | 0.181 | 0.042 |
| UCEC | -0.078 | 0.457 | -0.024 | 0.637 | -0.133 | 0.057 | -0.041 | 0.419 | 0.015 | 0.793 | 0.005 | 0.937 | -0.01 | 0.892 | -0.004 | 0.963 | 0.146 | 0.063 | 0.101 | 0.045 | -0.089 | 0.382 | -0.061 | 0.419 |
| UCS | -0.255 | 0.059 | -0.081 | 0.419 | -0.25 | 0.061 | -0.081 | 0.419 | -0.302 | 0.023 | -0.101 | 0.319 | -0.143 | 0.452 | -0.051 | 0.619 | -0.001 | 0.824 | -0.0004 | 0.981 | 0.08 | 0.598 | 0.021 | 0.781 |
| UVM | 0.373 | 0.001 | 0.121 | 0.087 | 0.325 | 0.003 | 0.112 | 0.121 | 0.339 | 0.002 | 0.112 | 0.121 | 0.426 | <0.001 | 0.151 | 0.087 | -0.052 | 0.684 | -0.019 | 0.819 | -0.088 | 0.524 | -0.031 | 0.748 |

DNAss, DNA methylation-based stemness index; EREG-METHss, epigenetically regulated DNA methylation-based stemness index; DMPss, differentially methylated probe-based stemness index; ENHss, enhancer element-based stemness index; RNAss, mRNA expression-based stemness index; EREG.EXPss, epigenetically regulated mRNA expression-based stemness index. Unadjusted: Spearman correlation. Adjusted: partial Spearman correlation controlling for M2 macrophage infiltration score (CIBERSORT). "cor" denotes the correlation coefficient; "p" denotes the two-sided P value.

**Table S7:** APOC1 clinical-relevant AS events on OncoSplicing

| Gene_Symbol | Splice_Type | Splice_Event | Project | Cancer_Type |
|---|---|---|---|---|
| APOC1 | AD | APOC1_AD_50358 | SpliceSeq | BLCA |
| APOC1 | AD | APOC1_AD_50360 | SpliceSeq | STAD/DLBC/KIRC/KIRP/UVM/TGCT/ UCEC/LIHC |
| APOC1 | AD | APOC1_AD_50361 | SpliceSeq | LIHC/THCA/SARC/LUSC/BRCA/ACC |
| APOC1 | AP | APOC1_AP_50356 | SpliceSeq | PRAD/UVM/ESCA/BRCA/KIRP/DLBC |
| APOC1 | AP | APOC1_AP_50357 | SpliceSeq | KIRP/PRAD/DLBC/BRCA/ESCA/UVM |
| APOC1 | ES | APOC1_ES_131923 | SpliceSeq | LIHC |
| APOC1 | ES | APOC1_ES_236364 | SpliceSeq | LIHC |
| APOC1 | ES | APOC1_ES_236370 | SpliceSeq | LIHC |
| APOC1 | ES | APOC1_ES_50359 | SpliceSeq | LGG/BLCA/THCA/KIRC/LAML/ESCA/ THYM/LIHC |
| APOC1 | ES | APOC1_ES_50362 | SpliceSeq | DLBCGBM/MESO/HNSC/ESCA |
| APOC1 | ES | APOC1_ES_50363 | SpliceSeq | ACC/BRCA/COAD/KIRC/THYM |
| APOC1 | ES | APOC1_ES_50364 | SpliceSeq | LGG/LIHC |
| APOC1 | ES | APOC1_ES_50365 | SpliceSeq | ACC/HNSC/LGG/LIHC |
| APOC1 | ES | APOC1_ES_50366 | SpliceSeq | BLCAHNSC/LGG/MESO |
| APOC1 | ES | APOC1_ES_50367 | SpliceSeq | COAD/DLBC/UCS |
| APOC1 | ES | APOC1_ES_50368 | SpliceSeq | CESC/LGG/HNSC/MESO/OV/ UVM |
| APOC1 | ES | APOC1_ES_50369 | SpliceSeq | ACC/BRCA/MESO/PRAD/UCEC/UVM/ GBM/OV/KICH |
| APOC1 | ES | APOC1_ES_50370 | SpliceSeq | UVM/LGG/LUAD/KIRP/THYM |
| APOC1 | ES | APOC1_ES_50371 | SpliceSeq | READ/CESC/BLCA/LIHC/LGG/UCEC/UVM |
| APOC1 | ES | APOC1_ES_99361 | SpliceSeq | ACC/LGG/LIHC/LUAD/KIRP |
| APOC1 | A3 | alt_3prime_129305 | SplAdder | LIHC |
| APOC1 | A3 | alt_3prime_129306 | SplAdder | ACC/KIRC/HNSC |
| APOC1 | A3 | alt_3prime_129312 | SplAdder | LUSC |
| APOC1 | A3 | alt_3prime_129313 | SplAdder | LIHC |
| APOC1 | A3 | alt_3prime_129331 | SplAdder | LIHC |
| APOC1 | A3 | alt_3prime_129334 | SplAdder | LIHC |
| APOC1 | A5 | alt_5prime_111741 | SplAdder | LIHC |
| APOC1 | A5 | alt_5prime_111758 | SplAdder | LIHC |
| APOC1 | A5 | alt_5prime_111760 | SplAdder | TGCT |
| APOC1 | A5 | alt_5prime_111763 | SplAdder | LIHC |
| APOC1 | A5 | alt_5prime_111766 | SplAdder | GBM/LIHC |
| APOC1 | A5 | alt_5prime_111768 | SplAdder | GBM/LUSC |
| APOC1 | A5 | alt_5prime_111769 | SplAdder | LIHC |
| APOC1 | A5 | alt_5prime_111782 | SplAdder | LIHC |
| APOC1 | A5 | alt_5prime_111797 | SplAdder | TGCT/LIHC/SARC/KIRP/LUAD/PAAD |
| APOC1 | A5 | alt_5prime_111799 | SplAdder | MESO/ACC/LIHC/KIRP/UVM/KIRC |
| APOC1 | A5 | alt_5prime_111804 | SplAdder | LIHC/TGCT |
| APOC1 | A5 | alt_5prime_111809 | SplAdder | LIHC |
| APOC1 | ES | exon_skip_308128 | SplAdder | LGG/UVM/MESO/KIRP/LIHC/ LUAD |
| APOC1 | ES | exon_skip_308131 | SplAdder | LIHC |
| APOC1 | ES | exon_skip_308136 | SplAdder | ACC/KIRC/TGCT |
| APOC1 | ES | exon_skip_308141 | SplAdder | ACC/LIHC |
| APOC1 | ES | exon_skip_308146 | SplAdder | LIHC |
| APOC1 | ES | exon_skip_308181 | SplAdder | READ |
| APOC1 | ES | exon_skip_308184 | SplAdder | HNSC/STAD/TGCT/MESO/LGG/UVM |
| APOC1 | ES | exon_skip_308186 | SplAdder | LUAD/THCA/UVM |
| APOC1 | ES | exon_skip_308189 | SplAdder | BRCA/TGCT/LUSC |
| APOC1 | ES | exon_skip_308192 | SplAdder | TGCT |
| APOC1 | ES | exon_skip_308195 | SplAdder | ACC/LGG/PAAD/MESO/KIRP |
| APOC1 | ES | exon_skip_308199 | SplAdder | LGG/CESC/MESO/ACC/PAAD/KIRP |
| APOC1 | ES | exon_skip_308202 | SplAdder | ACC |
| APOC1 | ES | exon_skip_308207 | SplAdder | ACC/MESO/PRAD/KIRP/CESC |
| APOC1 | ES | exon_skip_308209 | SplAdder | ACC/LGG/MESO/CESC/KIRP/ |

| Gene_Symbol | Splice_Type | Splice_Event | Project | Cancer_Type |
|---|---|---|---|---|
| | | | | PAAD |
| APOC1 | ES | exon_skip_308211 | SplAdder | CESC/MESO/ACC/KIRP/LGG/PAAD |
| APOC1 | ES | exon_skip_308212 | SplAdder | LIHC |
| APOC1 | ES | exon_skip_308214 | SplAdder | LIHC |
| APOC1 | ES | exon_skip_308216 | SplAdder | LIHC |
| APOC1 | ES | exon_skip_308217 | SplAdder | LIHC |
| APOC1 | ES | exon_skip_308235 | SplAdder | LIHC |
| APOC1 | ES | exon_skip_308242 | SplAdder | LIHC |
| APOC1 | ES | exon_skip_308243 | SplAdder | LIHC |
| APOC1 | ES | exon_skip_308245 | SplAdder | ACC/LIHC |
| APOC1 | ES | exon_skip_308246 | SplAdder | LIHC |
| APOC1 | ES | exon_skip_308247 | SplAdder | ACC |
| APOC1 | ES | exon_skip_308269 | SplAdder | LUSC |
| APOC1 | ES | exon_skip_308270 | SplAdder | LIHC |
| APOC1 | ES | exon_skip_308272 | SplAdder | LIHC |
| APOC1 | ES | exon_skip_308274 | SplAdder | KIRP/READ |
| APOC1 | ME | mutex_exons_3021696 | SplAdder | LIHC |
| APOC1 | ME | mutex_exons_3021700 | SplAdder | LIHC |
| APOC1 | ME | mutex_exons_3021702 | SplAdder | LIHC |
| APOC1 | ME | mutex_exons_3021704 | SplAdder | LIHC |
| APOC1 | ME | mutex_exons_3021705 | SplAdder | LIHC |
| APOC1 | ME | mutex_exons_3021706 | SplAdder | LIHC |
| APOC1 | ME | mutex_exons_3021707 | SplAdder | LIHC |
| APOC1 | ME | mutex_exons_3021720 | SplAdder | LIHC |
| APOC1 | ME | mutex_exons_3021730 | SplAdder | LIHC |
| APOC1 | ME | mutex_exons_3021731 | SplAdder | LIHC |
| APOC1 | ME | mutex_exons_3021732 | SplAdder | LIHC |
| APOC1 | ME | mutex_exons_3021734 | SplAdder | LIHC |
| APOC1 | ME | mutex_exons_3021735 | SplAdder | LIHC |
| APOC1 | ME | mutex_exons_3021739 | SplAdder | LIHC |
| APOC1 | ME | mutex_exons_3021740 | SplAdder | LIHC |
| APOC1 | ME | mutex_exons_3021743 | SplAdder | LIHC |
| APOC1 | ME | mutex_exons_3021744 | SplAdder | LIHC |
| APOC1 | ME | mutex_exons_3021745 | SplAdder | LIHC |
| APOC1 | ME | mutex_exons_3021746 | SplAdder | LIHC |
| APOC1 | ME | mutex_exons_3021748 | SplAdder | LIHC |
| APOC1 | ME | mutex_exons_3021749 | SplAdder | LIHC |
| APOC1 | ME | mutex_exons_3021750 | SplAdder | LIHC |
| APOC1 | ME | mutex_exons_3021752 | SplAdder | LIHC |
| APOC1 | ME | mutex_exons_3021753 | SplAdder | LIHC |
| APOC1 | ME | mutex_exons_3021754 | SplAdder | LIHC |
| APOC1 | ME | mutex_exons_3021757 | SplAdder | LIHC |
| APOC1 | ME | mutex_exons_3021759 | SplAdder | LIHC |
| APOC1 | ME | mutex_exons_3021761 | SplAdder | LIHC |
| APOC1 | ME | mutex_exons_3021781 | SplAdder | LIHC |
| APOC1 | ME | mutex_exons_3021785 | SplAdder | LIHC |
| APOC1 | ME | mutex_exons_3021788 | SplAdder | LIHC |
| APOC1 | ME | mutex_exons_3021791 | SplAdder | LIHC |
| APOC1 | ME | mutex_exons_3021792 | SplAdder | LIHC |

**Table S8:** GO pathways enriched by the top 100 APOC1 co-expressed genes identified on GEPIA2.0

| ONTOLOGY | ID | Description | GeneRatio | BgRatio | p.adjust | geneID | Count |
|---|---|---|---|---|---|---|---|
| BP | GO:0030193 | regulation of blood coagulation | 16/93 | 68/18870 | 4.58956610982846e-20 | PROC/VTN/F2/PLG/APOH/SERPINC1/KNG1/CPB2/SERPINF2/F12/F11/FGA/F7/KLKB1/APOE/HRG | 16 |
| BP | GO:1900046 | regulation of hemostasis | 16/93 | 70/18870 | 4.58956610982846e-20 | PROC/VTN/F2/PLG/APOH/SERPINC1/KNG1/CPB2/SERPINF2/F12/F11/FGA/F7/KLKB1/APOE/HRG | 16 |
| BP | GO:0007596 | blood coagulation | 22/93 | 224/18870 | 4.58956610982846e-20 | PROC/VTN/F2/PLG/APOH/C4BPB/SERPINC1/SERPINA10/KNG1/CPB2/SERPINF2/F12/SERPIND1/F11/F5/SERPINA1/FGA/F7/KLKB1/F13B/APOE/HRG | 22 |
| BP | GO:0050818 | regulation of coagulation | 16/93 | 73/18870 | 4.58956610982846e-20 | PROC/VTN/F2/PLG/APOH/SERPINC1/KNG1/CPB2/SERPINF2/F12/F11/FGA/F7/KLKB1/APOE/HRG | 16 |
| BP | GO:0050817 | coagulation | 22/93 | 229/18870 | 4.58956610982846e-20 | PROC/VTN/F2/PLG/APOH/C4BPB/SERPINC1/SERPINA10/KNG1/CPB2/SERPINF2/F12/SERPIND1/F11/F5/SERPINA1/FGA/F7/KLKB1/F13B/APOE/HRG | 22 |
| CC | GO:0072562 | blood microparticle | 30/94 | 144/19886 | 5.62993241344155e-40 | AMBP/ITIH1/APOA2/A1BG/ITIH4/VTN/F2/PLG/APOA1/SERPINC1/PON1/C8G/KNG1/TF/SERPINF2/CFHR1/HPX/C3/ALB/HP/ORM2/GC/C8A/HPR/FGA/AFM/APOE/CPN2/APCS/HRG | 30 |
| CC | GO:0062023 | collagen-containing extracellular matrix | 25/94 | 429/19886 | 5.86243509644794e-19 | AMBP/ITIH1/A1BG/ITIH4/VTN/F2/PLG/APOH/APOC3/APOA1/SERPINC1/KNG1/MST1/SERPINF2/F12/HPX/ORM2/ANGPTL3/SERPINA1/ANG/FGA/F7/APOE/APCS/HRG | 25 |
| CC | GO:0034358 | plasma lipoprotein particle | 12/94 | 36/19886 | 1.73057904396206e-18 | APOC1/APOC2/APOA2/APOH/APOC3/APOA1/PON1/APOB/SAA4/APOC4/HPR/APOE | 12 |
| CC | GO:1990777 | lipoprotein particle | 12/94 | 36/19886 | 1.73057904396206e-18 | APOC1/APOC2/APOA2/APOH/APOC3/APOA1/PON1/APOB/SAA4/APOC4/HPR/APOE | 12 |
| CC | GO:0034364 | high-density lipoprotein particle | 11/94 | 26/19886 | 2.11727229908251e-18 | APOC1/APOC2/APOA2/APOH/APOC3/APOA1/PON1/SAA4/APOC4/HPR/APOE | 11 |
| MF | GO:0004857 | enzyme inhibitor activity | 18/91 | 386/18496 | 1.06433475499711e-10 | APOC1/AMBP/APOC2/ITIH1/APOA2/ITIH4/APOC3/ITIH3/SERPINC1/SERPINA10/KNG1/SERPINF2/SERPIND1/C3/SERPINA4/ANGPTL3/SERPINA1/HRG | 18 |
| MF | GO:0004866 | endopeptidase inhibitor activity | 13/91 | 168/18496 | 1.49400302801793e-10 | AMBP/ITIH1/ITIH4/ITIH3/SERPINC1/SERPINA10/KNG1/SERPINF2/SERPIND1/C3/SERPINA4/SERPINA1/HRG | 13 |
| MF | GO:0004867 | serine-type endopeptidase inhibitor activity | 11/91 | 101/18496 | 1.49400302801793e-10 | AMBP/ITIH1/ITIH4/ITIH3/SERPINC1/SERPINA10/SERPINF2/SERPIND1/SERPINA4/SERPINA1/HRG | 11 |
| MF | GO:0008201 | heparin binding | 13/91 | 174/18496 | 1.49400302801793e-10 | VTN/F2/APOH/SERPINC1/SERPINA10/KNG1/SERPIND1/F11/APOB/ANGPTL3/ANG/APOE/HRG | 13 |
| MF | GO:0030414 | peptidase inhibitor activity | 13/91 | 175/18496 | 1.49400302801793e-10 | AMBP/ITIH1/ITIH4/ITIH3/SERPINC1/SERPINA10/KNG1/SERPINF2/SERPIND1/C3/SERPINA4/SERPINA1/HRG | 13 |

**Table S9:** List of abbreviations

| Full name | Abbreviations |
|---|---|
| Adrenocortical carcinoma | ACC |
| Bladder Urothelial Carcinoma | BLCA |
| Breast invasive carcinoma | BRCA |
| Cervical squamous cell carcinoma and endocervical adenocarcinoma | CESC |
| Cholangiocarcinoma | CHOL |
| Colon adenocarcinoma | COAD |
| Colon adenocarcinoma/Rectum adenocarcinoma Esophageal carcinoma | COADREAD |
| Lymphoid Neoplasm Diffuse Large B-cell Lymphoma | DLBC |
| Esophageal carcinoma | ESCA |
| FFPE Pilot Phase II | FPPP |
| Glioblastoma multiforme | GBM |
| Glioma | GBMLGG |
| Head and Neck squamous cell carcinoma | HNSC |
| Kidney Chromophobe | KICH |
| Pan-kidney cohort (KICH+KIRC+KIRP) | KIPAN |
| Kidney renal clear cell carcinoma | KIRC |
| Kidney renal papillary cell carcinoma | KIRP |
| Acute Myeloid Leukemia | LAML |
| Brain Lower Grade Glioma | LGG |
| Liver hepatocellular carcinoma | LIHC |
| Lung adenocarcinoma | LUAD |
| Lung squamous cell carcinoma | LUSC |
| Mesothelioma | MESO |
| Ovarian serous cystadenocarcinoma | OV |
| Pancreatic adenocarcinoma | PAAD |
| Pheochromocytoma and Paraganglioma | PCPG |
| Prostate adenocarcinoma | PRAD |
| Rectum adenocarcinoma | READ |
| Sarcoma | SARC |
| Stomach adenocarcinoma | STAD |
| Skin Cutaneous Melanoma | SKCM |
| Stomach and Esophageal carcinoma | STES |
| Testicular Germ Cell Tumors | TGCT |
| Thyroid carcinoma | THCA |
| Thymoma | THYM |
| Uterine Corpus Endometrial Carcinoma | UCEC |
| Uterine Carcinosarcoma | UCS |
| Uveal Melanoma | UVM |
| The Cancer Genome Atlas | TCGA |
| Genotype-Tissue Expression | GTEx |
| Clinical Proteomic Tumor Analysis Consortium | CPTAC |
| Gene Expression Omnibus | GEO |
| Cancer Cell Line Encyclopedia | CCLE |
| Chinese Glioma Genome Atlas | CGGA |
| International Cancer Genome Consortium | ICGC |
| European Genome-phenome Archive | EGA |
| Human Protein Atlas | HPA |
| cBioPortal for Cancer Genomics | cBioPortal |
| Tumor Immune Dysfunction and Exclusion | TIDE |
| Cancer Single-cell Expression Atlas | CancerSEA |
| The Cancer Proteome Atlas | TCPA |
| Overall Survival | OS |
| Disease-Specific Survival | DSS |
| Disease-Free Interval | DFI |
| Progression-Free Interval | PFI |
| Receiver Operating Characteristic | ROC |
| Area Under the Curve | AUC |
| Tumor Mutational Burden | TMB |
| Microsatellite Instability | MSI |

| **Full name** | **Abbreviations** |
|---|---|
| Mutant-Allele Tumor Heterogeneity | MATH |
| Homologous Recombination Deficiency | HRD |
| Loss of Heterozygosity | LOH |
| Single Nucleotide Variant | SNV |
| Percent Spliced In | PSI |
| Alternative Splicing | AS |
| Gene Set Enrichment Analysis | GSEA |
| single-sample GSEA | ssGSEA |
| Gene Ontology | GO |
| Kyoto Encyclopedia of Genes and Genomes | KEGG |
| Apolipoprotein C1 | APOC1 |
| Tumor-Associated Macrophages | TAMs |
| Cytotoxic T Lymphocyte | CTL |
| Epithelial-Mesenchymal Transition | EMT |
| Tumor Immune Microenvironment | TIME |
| DNA Mismatch Repair | MMR |
| Homologous Recombination Repair | HRR |
| DNA Methyltransferases | DNMTs |
| Histone Deacetylase | HDAC |
