# Supplementary material for: Pan-cancer analysis identifies APOC1 as a TAM-derived modulator of adaptive immune resistance and predictor of therapeutic response
Source: Discov Oncol. 2026 Jun 25;17:1317. doi: 10.1007/s12672-026-05515-x (PMC13554021; doi:10.1007/s12672-026-05515-x)
Supplement: Supplementary file 2 [file 12672_2026_5515_MOESM2_ESM.pdf]

Supplementary material 1

Supplementary figures for: Pan-Cancer Analysis Identifies APOC1 as a TAM-Derived Modulator of Adaptive Immune Resistance and Predictor of Therapeutic Response

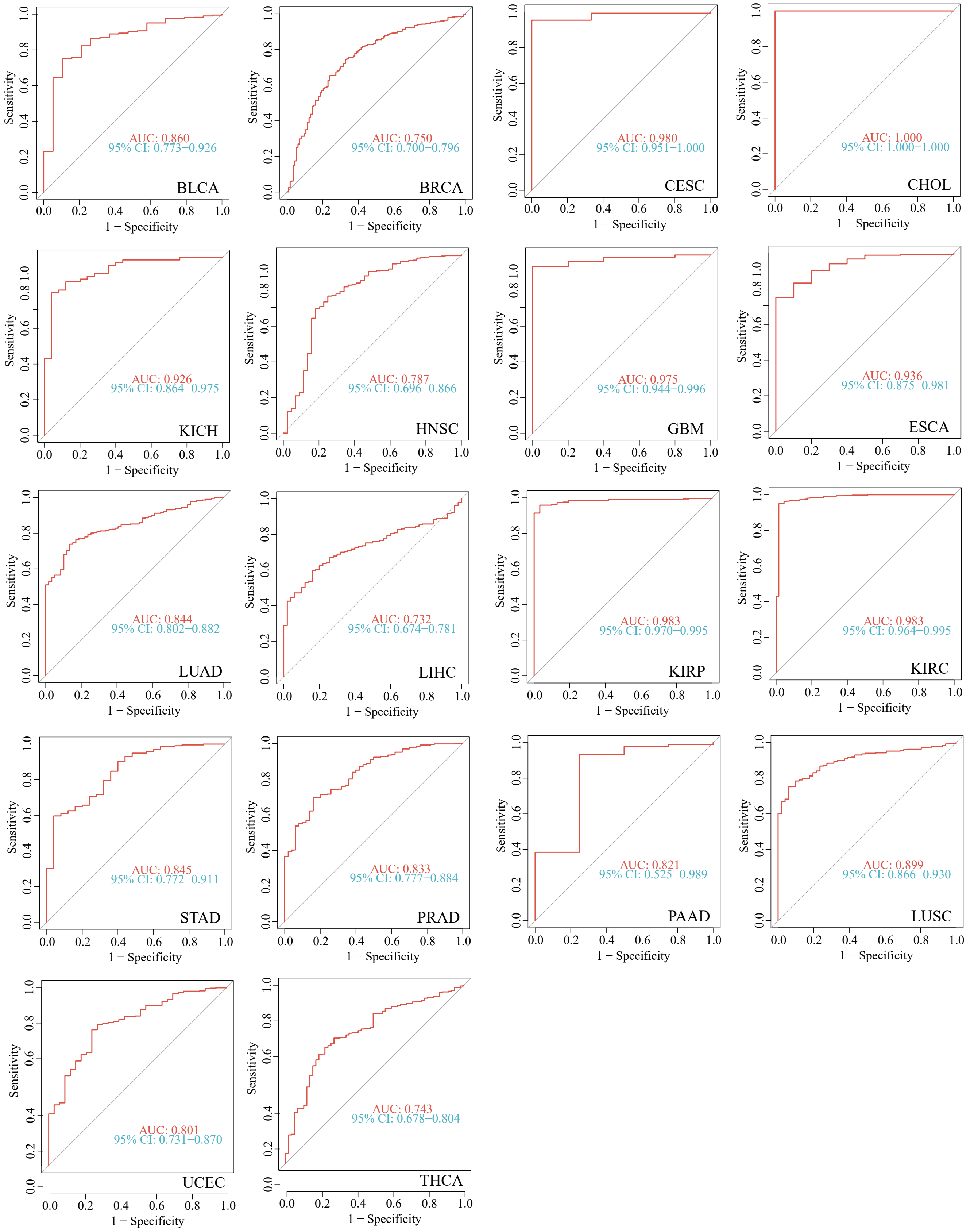

Figure S1: Diagnostic Value of APOC1 Across Cancers. ROC curves and AUC values for APOC1 expression in distinguishing tumor from normal tissues in various cancer types, based on integrated TCGA and GTEx data.

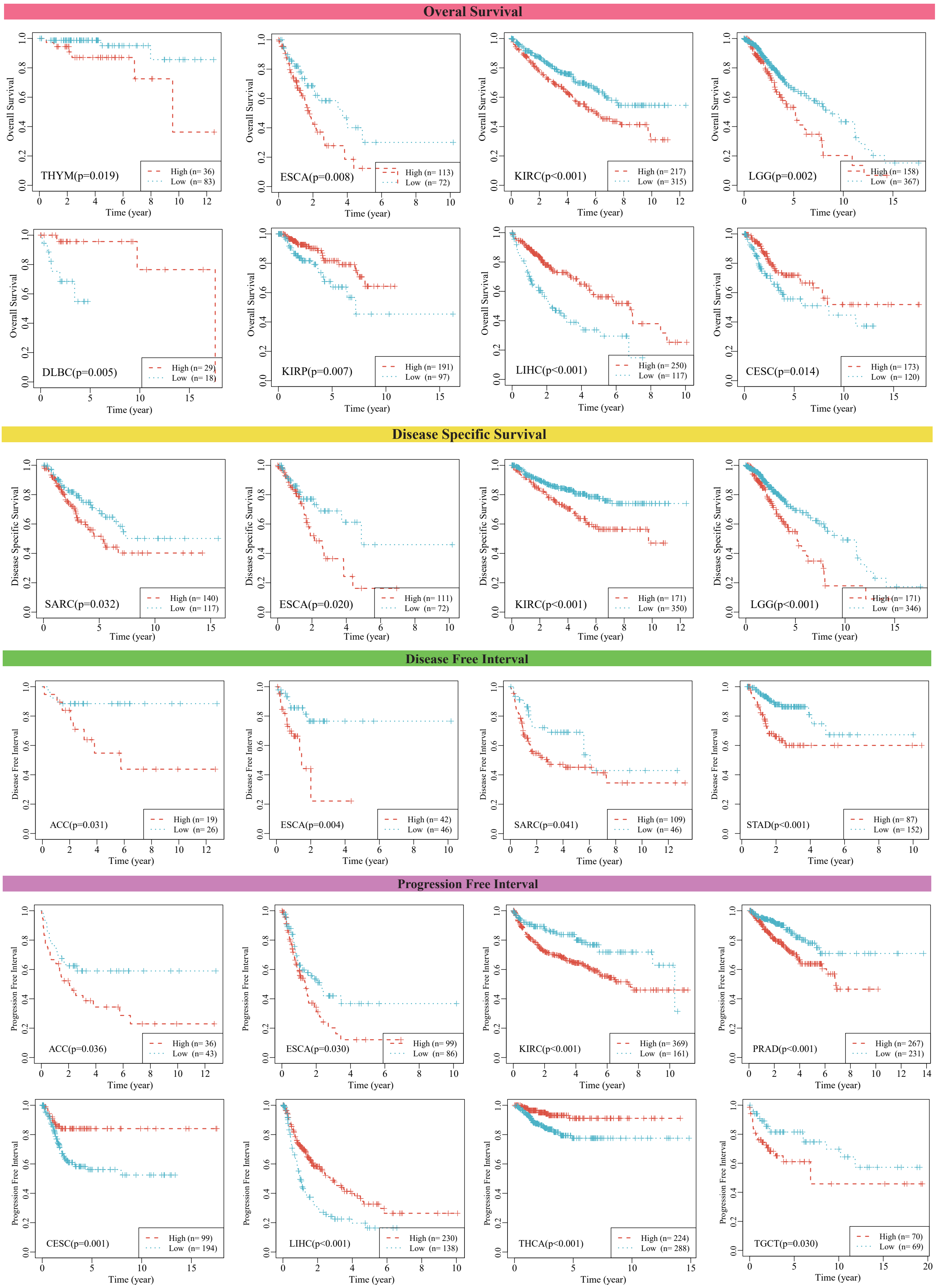

Figure S2: Kaplan-Meier Survival Curves for APOC1 in Representative Cancers

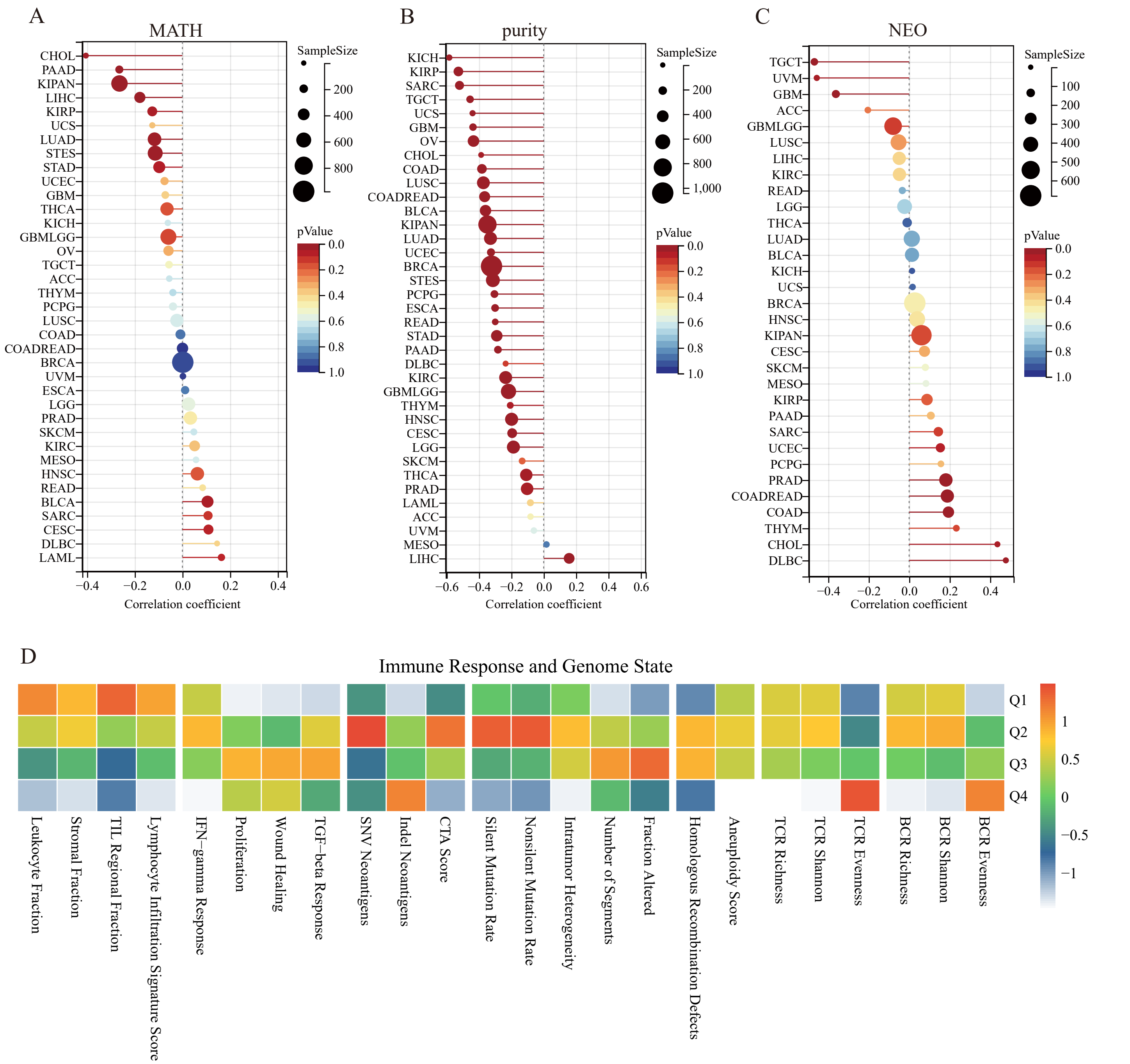

Figure S3: Correlation of APOC1 with Additional Genomic Instability Markers. **(A-C)** Correlation between APOC1 expression and MATH score **(A)**, tumor purity **(B)**, and NEO across **(C)** cancer types. **(D)** Correlation between APOC1 expression and immune-related genes in pan-cancer

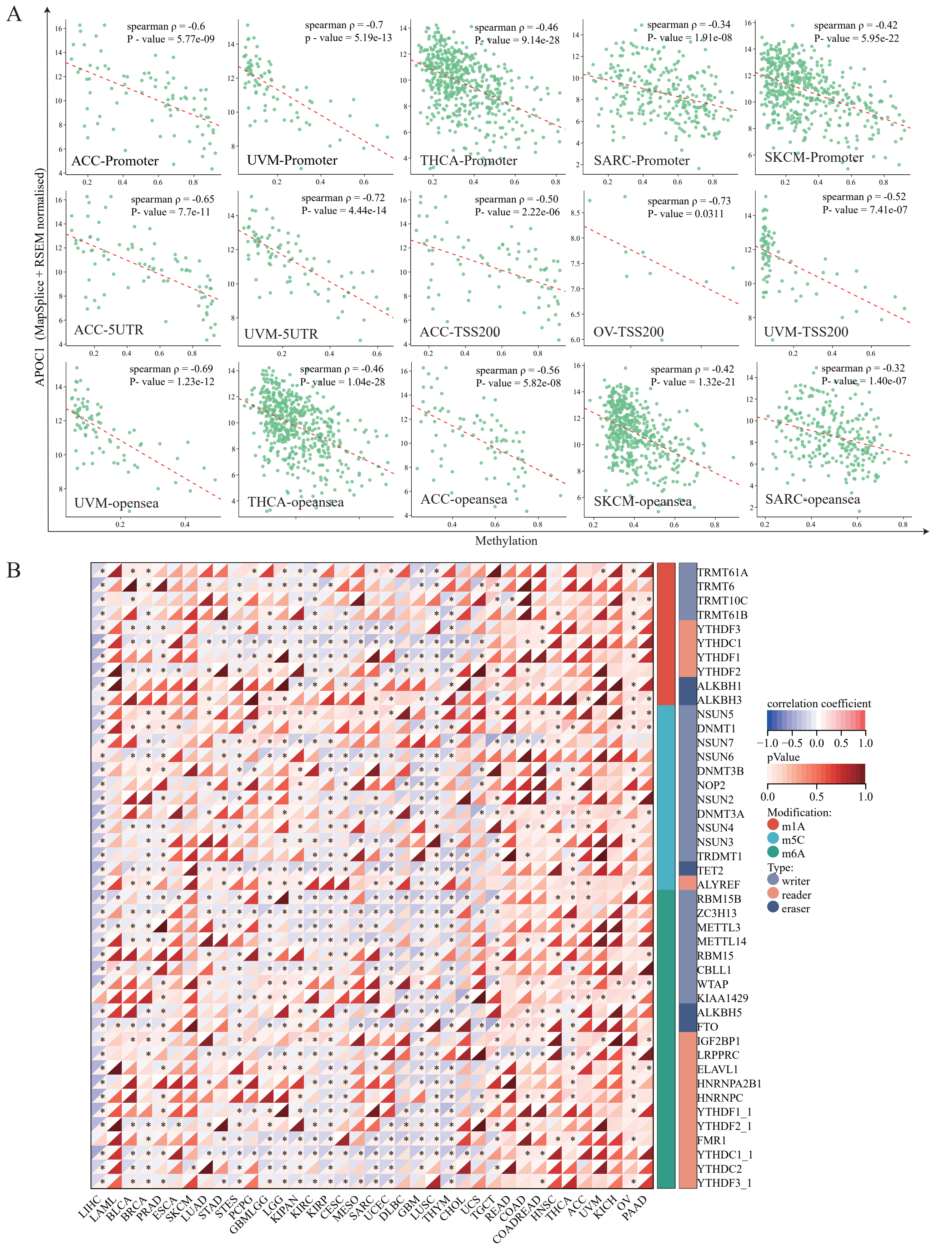

Figure S4: Correlation Between APOC1 Expression and Epigenetic Regulatory Networks. **(A)** Spearman correlation between APOC1 mRNA expression and its own promoter methylation levels across cancer types. **(B)** Heatmap showing Spearman correlation between APOC1 expression and 44 m^1A, m^5C, and m^6A-related epitranscriptomic modifiers across cancer types.

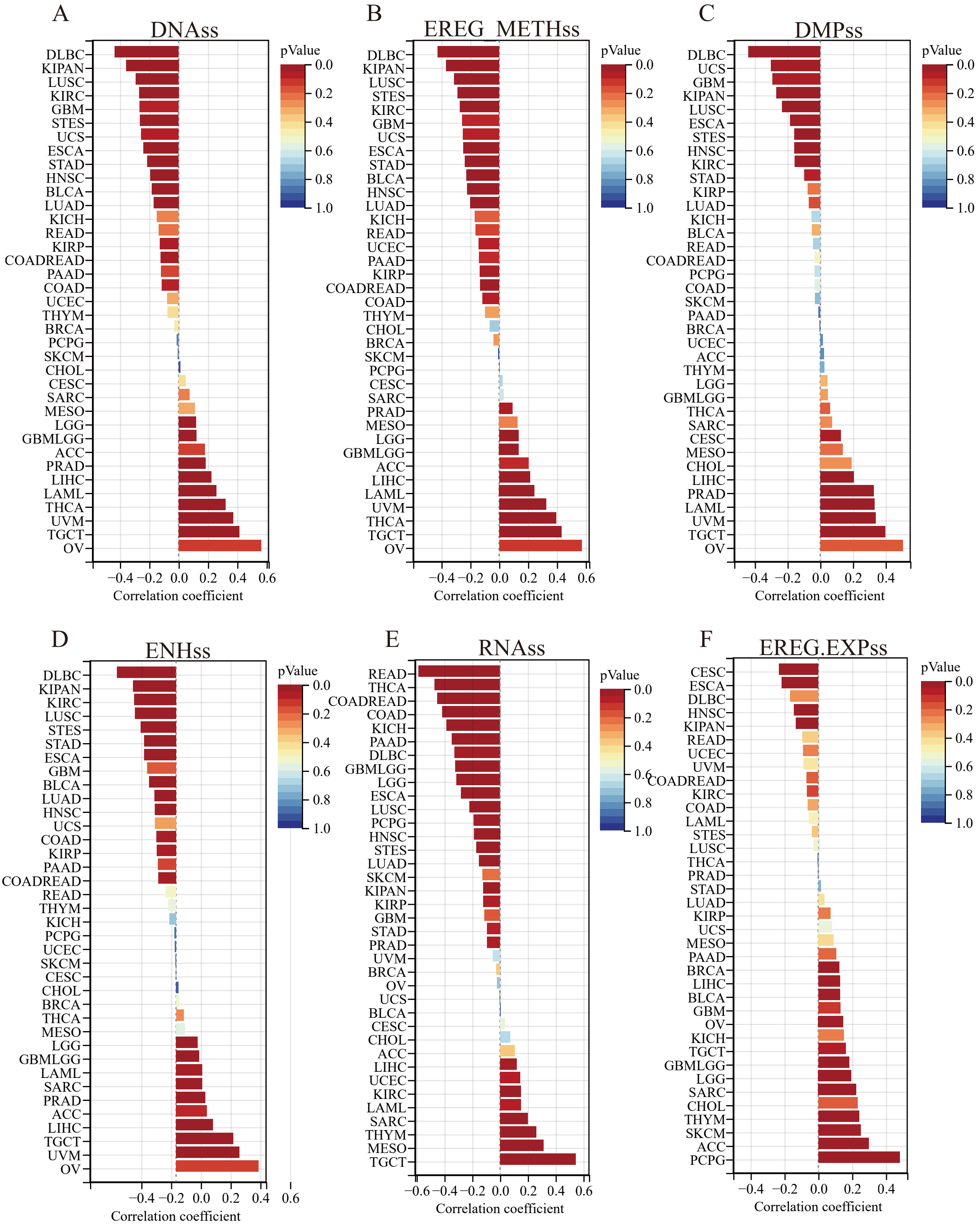

Figure S5: Correlation Between APOC1 Expression and Tumor Stemness Indices Across Cancers. **(A-D)** Spearman correlation between APOC1 expression and DNA methylation-based stemness indices across cancer types: DNAss **(A)**, EREG-METHss **(B)**, DMPss **(C)**, and ENHss **(D)**. **(E-F)** Spearman correlation between APOC1 expression and mRNA expression-based stemness indices across cancer types: RNAss **(E)** and EREG.EXPss **(F)**.

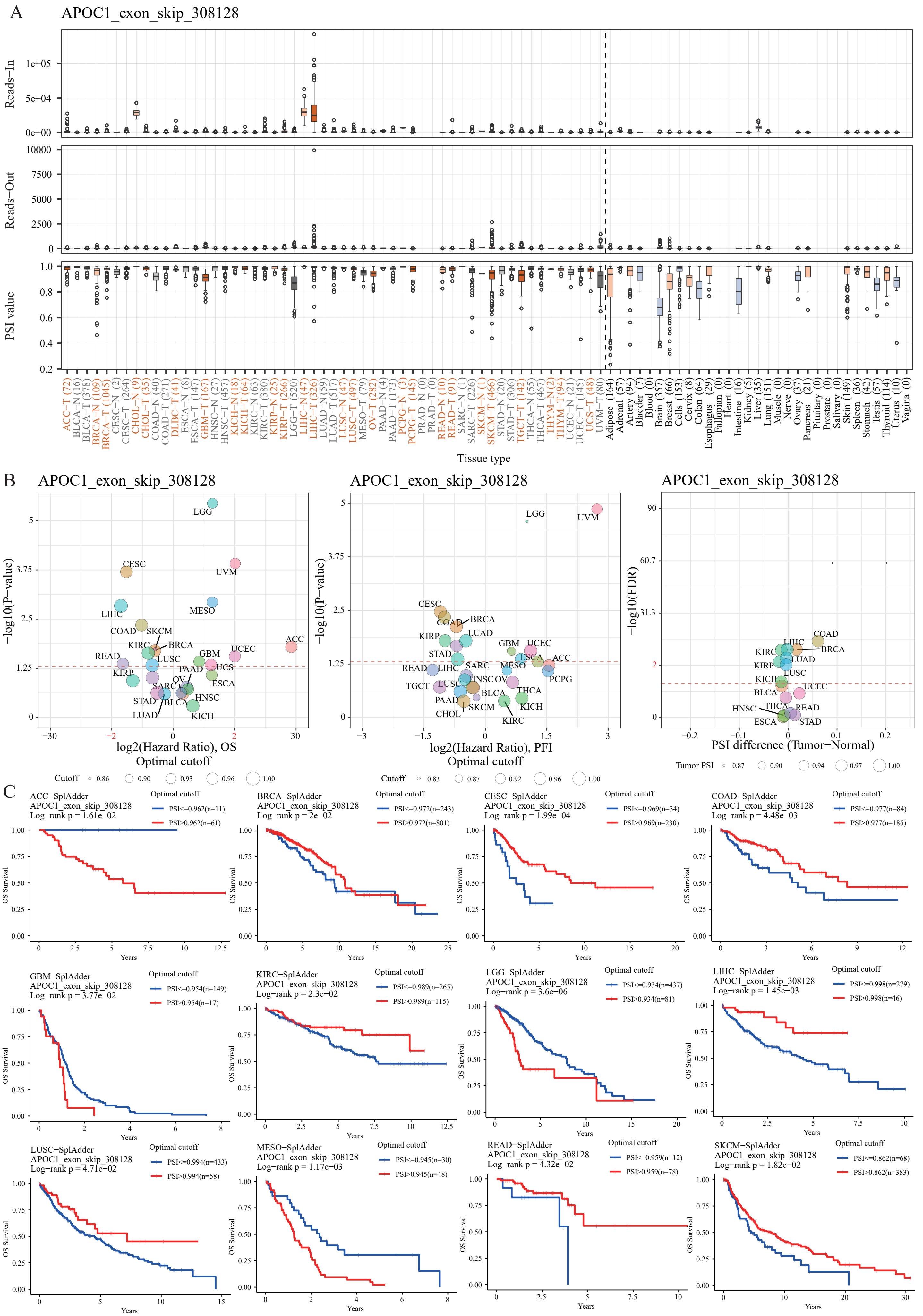

Figure S6: Clinical Relevance of APOC1 Exon Skip Event APOC1_exon_skip_308128. **(A)** Comparison of percent spliced index (PSI) values for APOC1_exon_skip_308128 between tumor and normal tissues across cancer types. **(B)** Correlation between APOC1_exon_skip_308128 PSI values and patient prognosis. **(C)** Kaplan-Meier survival curves from OncoSplicing showing the prognostic significance of APOC1_exon_skip_308128 PSI levels in selected cancer types.

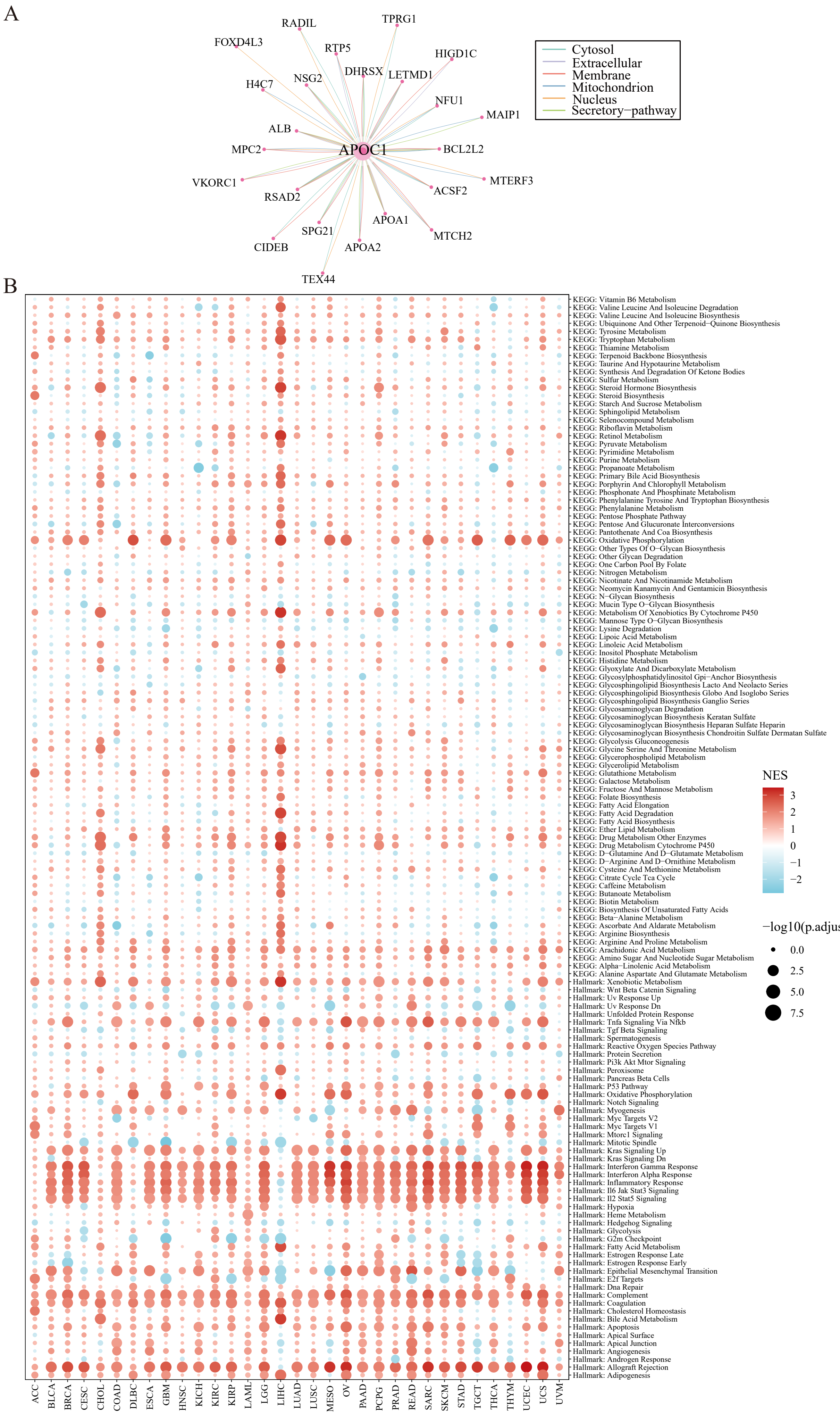

Figure S7: Protein Interaction Network and GSEA Enrichment Analysis of APOC1. **(A)** Protein-protein interaction (PPI) network of APOC1 from the Comppi database, filtered for proteins sharing common subcellular localization. **(B)** GSEA plots showing enrichment of immune-related Hallmark pathways (inflammatory response, epithelial-mesenchymal transition, interferon-gamma response, complement) in APOC1 high-expression samples compared to low-expression samples.

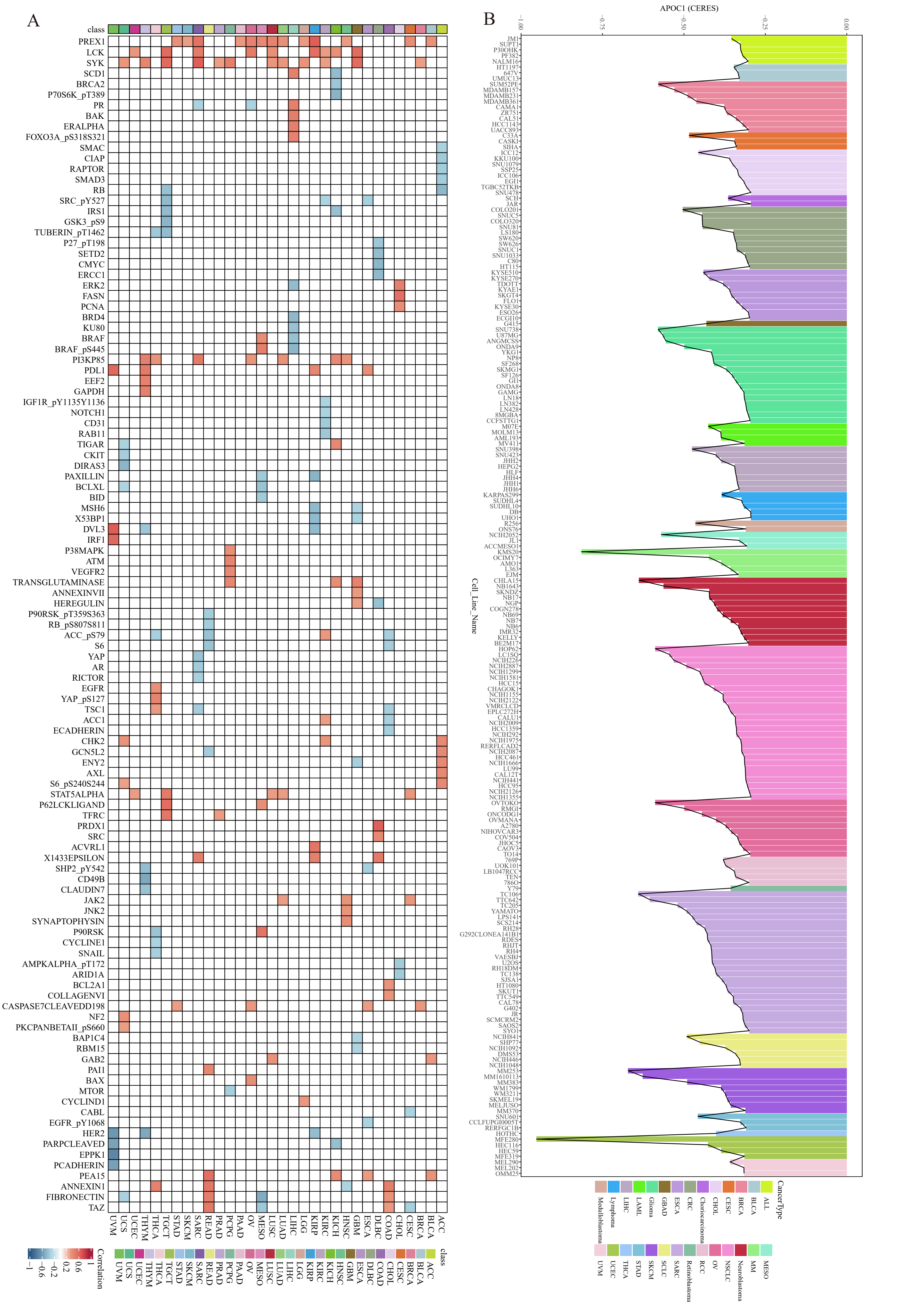

Figure S8: Protein-Level Correlations and APOC1 Dependency Across Cancer Cell Lines. **(A)** Correlation between APOC1 expression and protein levels of top positively and negatively correlated proteins from RPPA data across cancer types. **(B)** Distribution of APOC1 CERES dependency scores across cancer cell lines from the DepMap database. Lower CERES scores indicate stronger proliferative inhibitory effect upon APOC1 knockout.

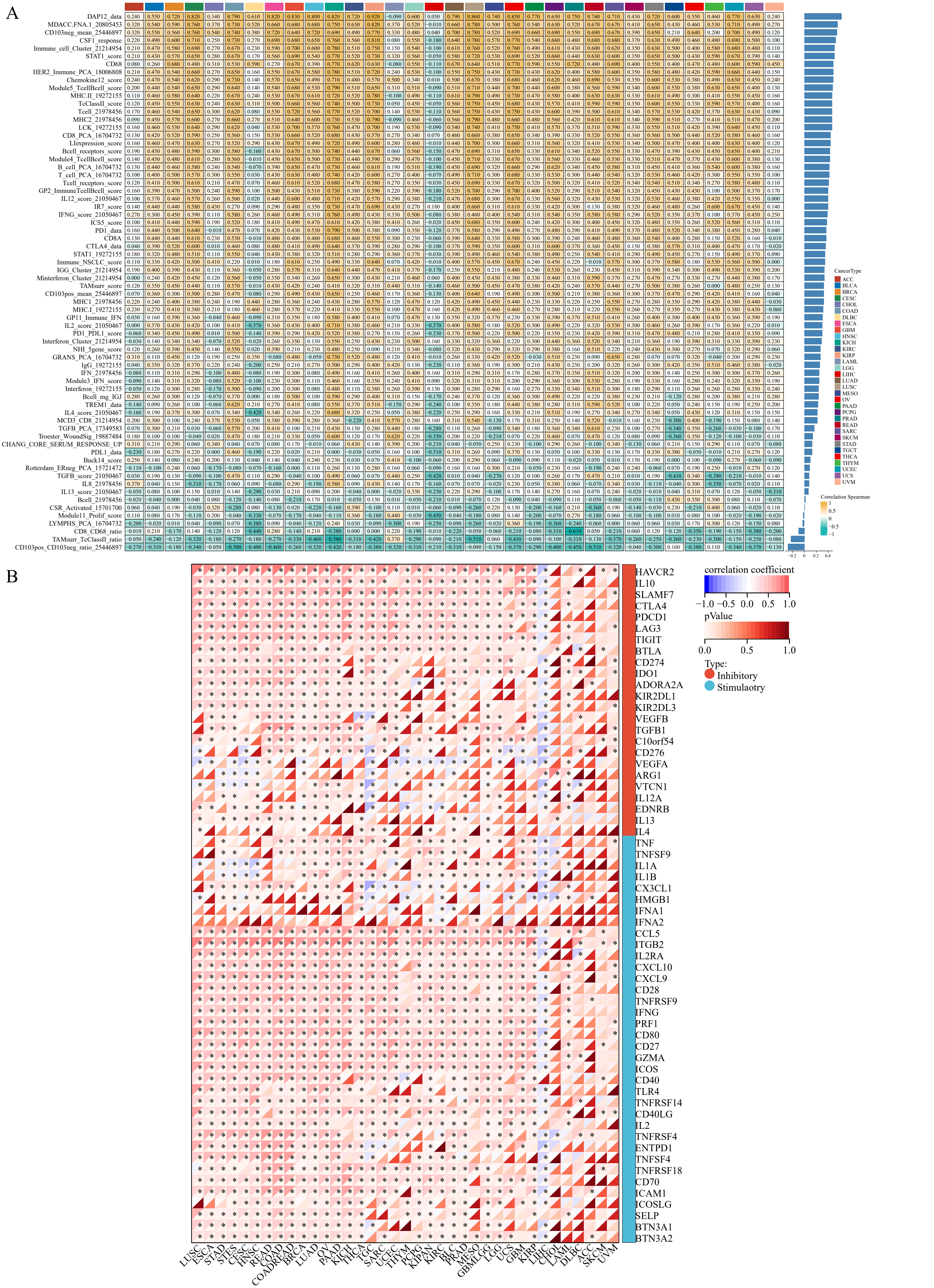

Figure S9: Correlation Between APOC1 Expression and Immune-Related Signatures and Checkpoint Molecules. **(A)** Heatmap depicting correlations between APOC1 expression and 68 immune-related signatures (encompassing immune cell infiltration, inflammation, antigen presentation, and cytokine signaling) across cancer types. **(B)** Heatmap of correlations between APOC1 expression and 60 immune checkpoint molecules (24 inhibitory, 36 stimulatory) across cancer types. *p < 0.05, **p < 0.01, ***p < 0.001.

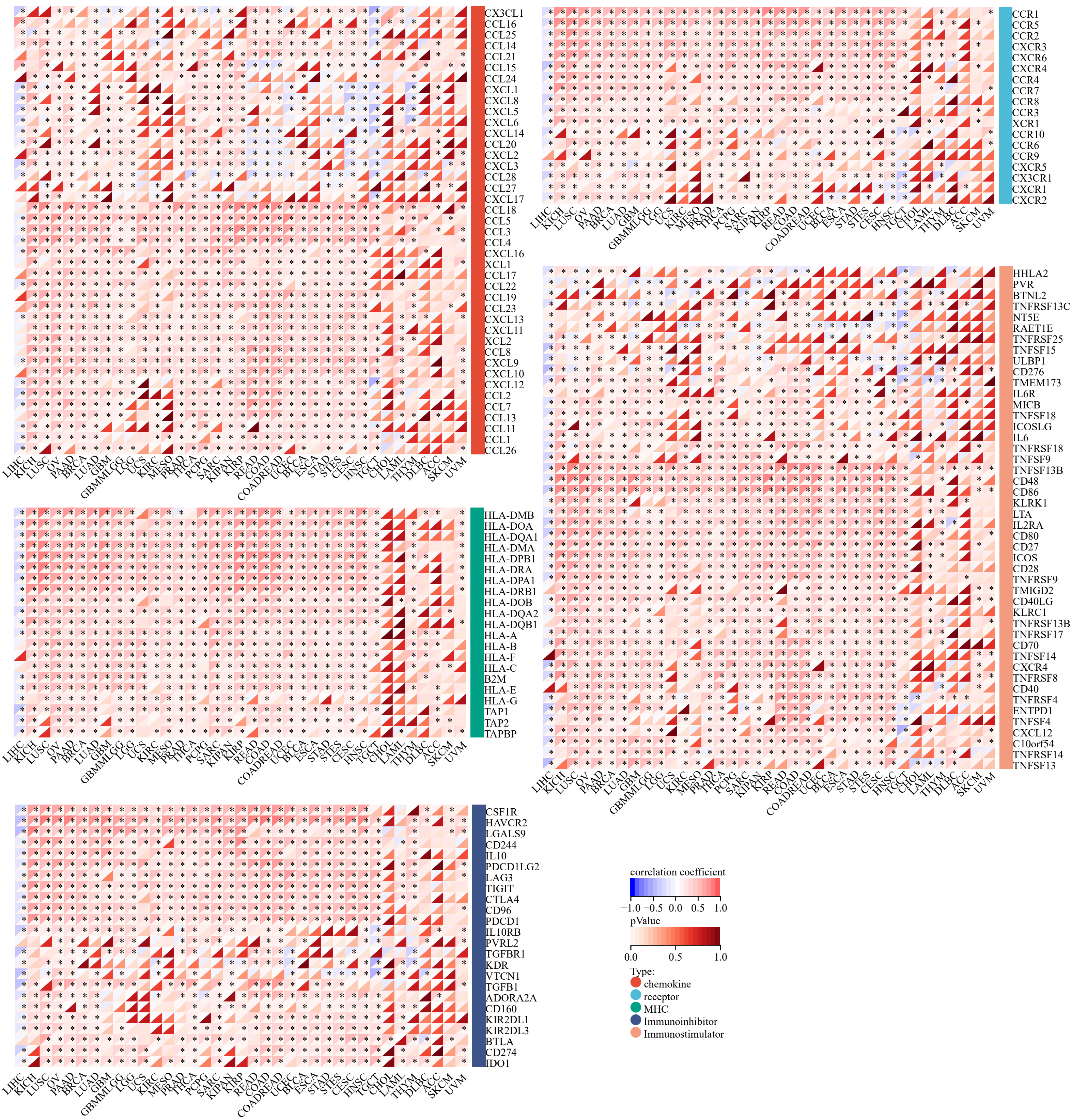

Figure S10: Comprehensive Characterization of APOC1 in Immune Regulation. Heatmap of Spearman correlations between APOC1 expression and 150 immune modulator genes across cancer types, showing tumor-dependent association patterns.
